# Genome-wide association mapping in a sweet cherry germplasm collection (*Prunus avium* L.) reveals candidate genes for fruit quality traits

**DOI:** 10.1093/hr/uhad191

**Published:** 2023-09-19

**Authors:** Armel S L Donkpegan, Anthony Bernard, Teresa Barreneche, José Quero-García, Hélène Bonnet, Mathieu Fouché, Loïck Le Dantec, Bénédicte Wenden, Elisabeth Dirlewanger

**Affiliations:** UMR BFP, INRAE, University of Bordeaux, 71 Avenue Edouard Bourlaux, F-33882 Villenave d’Ornon, France; UMR BOA, SYSAAF, Centre INRAE Val de Loire, 37380 Nouzilly, France; UMR BFP, INRAE, University of Bordeaux, 71 Avenue Edouard Bourlaux, F-33882 Villenave d’Ornon, France; UMR BFP, INRAE, University of Bordeaux, 71 Avenue Edouard Bourlaux, F-33882 Villenave d’Ornon, France; UMR BFP, INRAE, University of Bordeaux, 71 Avenue Edouard Bourlaux, F-33882 Villenave d’Ornon, France; UMR BFP, INRAE, University of Bordeaux, 71 Avenue Edouard Bourlaux, F-33882 Villenave d’Ornon, France; UMR BFP, INRAE, University of Bordeaux, 71 Avenue Edouard Bourlaux, F-33882 Villenave d’Ornon, France; UMR BFP, INRAE, University of Bordeaux, 71 Avenue Edouard Bourlaux, F-33882 Villenave d’Ornon, France; UMR BFP, INRAE, University of Bordeaux, 71 Avenue Edouard Bourlaux, F-33882 Villenave d’Ornon, France; UMR BFP, INRAE, University of Bordeaux, 71 Avenue Edouard Bourlaux, F-33882 Villenave d’Ornon, France

## Abstract

In sweet cherry (*Prunus avium* L.), large variability exists for various traits related to fruit quality. There is a need to discover the genetic architecture of these traits in order to enhance the efficiency of breeding strategies for consumer and producer demands. With this objective, a germplasm collection consisting of 116 sweet cherry accessions was evaluated for 23 agronomic fruit quality traits over 2–6 years, and characterized using a genotyping-by-sequencing approach. The SNP coverage collected was used to conduct a genome-wide association study using two multilocus models and three reference genomes. We identified numerous SNP–trait associations for global fruit size (weight, width, and thickness), fruit cracking, fruit firmness, and stone size, and we pinpointed several candidate genes involved in phytohormone, calcium, and cell wall metabolisms. Finally, we conducted a precise literature review focusing on the genetic architecture of fruit quality traits in sweet cherry to compare our results with potential colocalizations of marker–trait associations. This study brings new knowledge of the genetic control of important agronomic traits related to fruit quality, and to the development of marker-assisted selection strategies targeted towards the facilitation of breeding efforts.

## Introduction

Sweet cherry (*Prunus avium* L.), a diploid species (2*n* = 2*x* = 16 chromosomes), is one of the most economically important perennial fruit species growing in temperate regions [1, 2]. The world production of sweet cherries has increased in the last two decades to reach 2.5 million tons per year (FAOSTAT, http://faostat.fao.org/, 2022). The top producers are Turkey, USA, Chile, China, and Iran, and production in Europe is dominated by Italy and Spain, followed by France. However, the general trend in cherry production in France has been declining markedly since the 1980s, from 112 000 in 1980 to 35 000 tons per year nowadays, partly due to climate change impacts, the general drop in agricultural production, and social change, as well as increased biotic pressure from pathogens such as the fruit fly *Drosophila suzukii* [3].

For consumers, important sweet cherry quality traits are large fruit, light or dark red skin color, the balance between sugar and acid content, and flesh firmness and low cracking susceptibility [4]. In addition, for producers, resistance to fruit cracking due to rainfall in spring is particularly important as it may be responsible for high production losses [5–8]. However, the creation of new sweet cherry cultivars from traditional breeding is still a long process, because of the extended juvenile phase, the size of the area required for evaluation, and complex polygenic traits [9].

In the last decade, sweet cherry breeders have investigated fruit quality selection, mostly using quantitative trait locus (QTL) mapping detection in biparental populations. QTLs have been detected for fruit firmness, with major loci on chromosomes 1 and 4 [10–14], global fruit size including fruit weight and fruit diameter [11–13, 15–17], and fruit cracking [18], together with other important agronomic traits related to phenology, such as flowering date [19, 20] and maturity date [13, 20, 21].

However, QTL detection in biparental populations can suffer from limitations [22] and success in finding QTLs depends on several elements, such as the heterozygosity level of the two parents of the progeny and the phenotypic variability of the trait studied between the two parents. Fortunately, several studies with the use of many years of data have allowed a significant reduction of QTL confidence intervals for traits of interest in sweet cherry [11, 19]. Genome-wide association study (GWAS) is complementary to QTL mapping and includes the genetic diversity of individuals with little or no familial relatedness [23]. However, association mapping has some crucial factors to be accounted for, such as the need for high-density genotyping data, the level of linkage disequilibrium (LD), the phenotypic variability of the individuals represented within the population, the population structure and kinship, the complexity of the trait, and the inclusion of rare alleles, all conditioning the detection of marker–trait associations [24, 25]. In sweet cherry, GWAS was recently implemented to characterize the genetic architecture of fruit-size-related traits, which confirmed the previous locus involved in fruit firmness on chromosome 4 detected by linkage mapping [26].

New approaches, such as genotyping-by-sequencing (GBS), allow the sequencing of many samples at the same time and can be used to obtain high-density sets of SNPs at a lower cost [27]. During recent years, the usefulness of GBS in perennial tree fruit species has been well documented [28–31], including the creation of a high-density linkage map in *Prunus* species [32, 33] and GWAS in peach [34, 35]. These GBS approaches in *Prunus* species have been made possible thanks to the availability of peach [36] and sweet cherry [37, 38] reference genomes.

In the present work, we report the results of a GWAS for fruit quality traits in a germplasm collection of sweet cherry accessions genotyped using the GBS approach. Specifically, the main objectives of this study were to (i) examine the existing variability of 23 fruit quality traits within a sample of 116 genotypes of sweet cherry, (ii) identify genome-wide SNP markers associated with these traits by GWAS using three reference genomes and two mixed linear models, (iii) compare our results with those previously reported, and (iv) investigate the putative functions, based on searches for orthologs, of the candidate genes associated with these SNPs.

## Results

### Phenotypic variation of sweet cherry fruit quality traits

**Figure 1:**
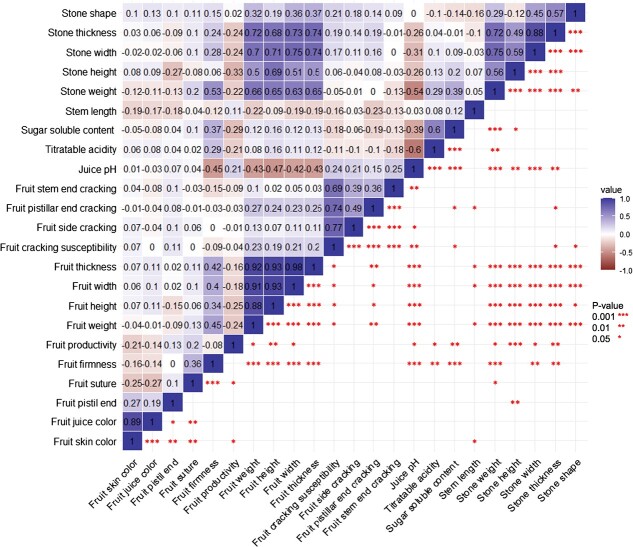
Pearson correlation matrix of the 23 studied traits.

For each trait, descriptive statistics, including minimum and maximum values, means and standard deviations, are given in Table 1. A high phenotypic variation was observed for most of the 23 fruit quality traits in the 116 accessions. For instance, concerning fruit-related traits, fruit weight (FW) varied from 1.70 to 16.61 g depending on the year and the accession. On the contrary, pH had a low range of variation, from 2.93 to 4.04 in 2015 (Table 1). As expected, strong positive correlations were found between fruit-related traits [FW, fruit height (FH), fruit thickness (FT), and fruit width (FWi)], and extended from 0.88 to 0.98 (Fig. 1). The same was true between stone-related traits [stone weight (SW), stone height (SH), stone thickness (ST), and stone width (SWi)], which were positively correlated (0.49–0.88). Fruit-related traits were also positively correlated with stone-related traits, indicating that a bigger fruit tends to have a bigger stone. Fruit-cracking-related traits were slightly positively correlated (from 0.36 to 0.49); on the contrary, pH tended to be negatively correlated with both fruit- and stone-related traits.

**Table 1:** Descriptive statistics and broad-sense heritabilities (*H*^2^) for the 23 traits evaluated during 2–6 years.

**Trait**	**Unit** ^**a**^	**Year**	**Mean ± SD**	**Range**	*H* ^2^
**Fruit**					
Fruit skin color		2014	5.55 ± 1.57	1–9	0.90
		2015	5.37 ± 1.53	1–9	
Fruit juice color		2014	4.65 ± 2.03	1–9	0.86
		2015	4.36 ± 1.79	1–9	
Fruit pistil end		2014	2.39 ± 0.70	1–3	0.89
		2015	2.36 ± 0.71	1–3	
Fruit suture		2014	2.08 ± 0.60	1–3	0.29
		2015	1.82 ± 0.71	1–3	
Fruit firmness		2014	60.70 ± 10.20	26–83	0.90
		2015	67.23 ± 11.43	29–95	
		2016	72.11 ± 11.71	36.55–102.94	
		2017	63.38 ± 9.71	32.65–91.90	
		2018	60.61 ± 10.97	27.55–88.40	
		2019	64.27 ± 10.94	30.25–94.05	
Fruit productivity		2014	5.56 ± 1.46	2–9	0.63
		2015	3.73 ± 1.57	1–8	
		2016	4.24 ± 1.72	0–8	
		2017	3.62 ± 1.80	0–8	
		2018	3.49 ± 1.53	1–7	
		2019	4.73 ± 1.84	1–9	
Fruit weight	g	2014	5.27 ± 1.65	1.70–10.26	0.92
		2015	5.45 ± 1.53	2.08–11.29	
		2016	6.21 ± 1.70	2.20–16.61	
		2017	5.73 ± 1.55	2.15–10.04	
		2018	6.05 ± 1.65	2.62–11.39	
		2019	5.36 ± 1.54	1.87–9.85	
Fruit height	mm	2014	18.31 ± 1.97	12.76–23.60	0.86
		2015	19.14 ± 2.04	12.91–24.34	
Fruit width (FWi)	mm	2014	17.05 ± 1.89	11.45–22.47	0.81
		2015	21.13 ± 2.50	14.29–27.94	
Fruit thickness (FT)	mm	2014	19.59 ± 2.31	12.41–26.50	0.84
		2015	18.72 ± 2.18	2.691–23.81	
Fruit cracking susceptibility (FCS)		2014	2.88 ± 1.64	1–9	0.63
		2015	1.27 ± 0.45	1–3	
		2016	3.08 ± 1.60	1–9	
		2017	1.93 ± 0.93	1–7	
		2018	3.83 ± 1.72	1–9	
		2019	2.03 ± 1.03	1–9	
Fruit side cracking (FS)	%	2014	0.07 ± 0.08	0–0.76	0.36
		2015	0.0025 ± 0.005	0–0.06	
		2016	0.04 ± 0.05	0–0.34	
		2017	0.01 ± 0.02	0–0.12	
		2018	0.08 ± 0.07	0–0.38	
		2019	0.03 ± 0.04	0–0.78	
Fruit pistillar end cracking (PI)	%	2014	0.05 ± 0.07	0–0.64	0.64
		2015	0.006 ± 0.011	0–0.22	
		2016	0.03 ± 0.04	0–0.38	
		2017	0.00 ± 0.00	0–0	
		2018	0.07 ± 0.08	0–0.42	
		2019	0.005 ± 0.009	0–0.3	
Fruit stem end cracking	%	2014	0.04 ± 0.05	0–0.52	0.58
		2015	0.007 ± 0.012	0–0.18	
		2016	0.11 ± 0.13	0–0.86	
		2017	0.03 ± 0.04	0–0.26	
		2018	0.07 ± 0.07	0–0.58	
		2019	0.02 ± 0.03	0–0.4	
**Fruit juice**					
Juice pH		2014	3.63 ± 0.18	3.17–4.25	0.75
		2015	3.54 ± 0.18	2.93–4.04	
Titratable acidity	mEq/100 ml	2014	12.12 ± 2.11	4.51–21.45	0.49
		2015	10.12 ± 2.10	6.21–22.49	
Soluble sugar content	% Brix	2014	14.23 ± 2.13	8.9–20.00	0.31
		2015	15.63 ± 2.49	8.8–23.40	
**Stem**					
Stem length	mm	2014	33.47 ± 5.18	20.80–55.00	0.87
		2015	35.83 ± 5.96	20.30–53.50	
**Stone**					
Stone weight	g	2014	0.23 ± 0.05	0.05–0.41	0.80
		2015	0.25 ± 0.05	0.08–0.38	
Stone height	mm	2014	10.45 ± 0.74	8.19–12.99	0.87
		2015	10.46 ± 0.68	8.41–12.37	
Stone width	mm	2014	8.47 ± 0.58	5.99–10.19	0.83
		2015	8.70 ± 0.57	6.64–9.95	
Stone thickness	mm	2014	6.73 ± 0.59	4.65–9.40	0.86
		2015	7.00 ± 0.55	4.99–8.44	
Stone shape in central view		2014	1.93 ± 0.16	1–3	0.46
		2015	1.96 ± 0.55	1–3	

Broad-sense heritability (*H*^2^) was high for most of the traits (Table 1). For instance, *H*^2^ varied from 0.81 to 0.92 for traits related to fruit size (FW, FH, FWi, and FT). Cracking-related traits, however, tended to be less inheritable (between 0.36 and 0.64), as were juice-related traits (between 0.31 and 0.75). The least inheritable trait was fruit suture (FSU), with *H*^2^ = 0.29. Some traits did not show a normal distribution (Supplementary Data Fig. S1). For example, cracking-related traits had a left-skewed distribution, and both fruit juice color (FJC) and fruit pistil end (FPE) had a non-normal distribution.

### SNP discovery through genotyping-by-sequencing

Sequencing of the prepared GBS libraries of the panel resulted in 389 682 215 raw reads with an average of 7.9 million reads per sample (Supplementary Data Fig. S2), which were aligned to three reference genomes. Alignment on the *P. avium* var. ‘Regina’ reference genome and variant calling yielded a total of 574 556 SNPs (Supplementary Data Table S1). After removing SNPs based on minimum quality, depth, and <20% missing data to ensure a high-quality SNP set for GWAS, we retained 75 916 SNPs. After filtering for >5% minor allele frequency (MAF), GWAS was conducted with 28 198 SNPs. The same filtering method was applied after alignment on *P. avium* var. ‘Satonishiki’ and *Prunus persica* ‘PLov2-2n’ genomes, and we retained 34 864 and 33 760 SNPs, respectively (Supplementary Data Table S1).

### Population structure and linkage disequilibrium

Based on a cross-validation procedure and considering the grouping threshold of Q > 0.70 in structure analyses, the most likely number of subpopulations depended on the SNP dataset used: *K* = 10, *K* = 9, and *K* = 6 for the GBS sequences aligned to the ‘Regina’ genome (Supplementary Data Fig. S3-a1), ‘Satonishiki’ genome (Supplementary Data Fig. S3-b1), and ‘PLov2-2n’ genome (Supplementary Data Fig. S3-c1), respectively. The six subpopulations found using the SNP obtained with the ‘PLov2-2n’ genome were also detected with the two other SNP datasets, although they were not strictly identical (Supplementary Data Fig. S3a-2, b-2, and c-2). For instance, the subpopulation (colored in yellow in the Fig. S3) that consisted of 12 accessions when using the SNP dataset obtained with the ‘Regina’ genome were all found within the corresponding subpopulations identified when using SNPs from ‘Satonishiki’ (19 accessions) and SNPs from ‘PLov2-2n’ (34 accessions). Overall, a large proportion of genotypes were classified as admixed (e.g. 59% using SNPs obtained from ‘Regina’). The different subgroups identified in structure analyses including the admixed genotypes were projected onto the first two components of the principal component analysis (PCA) performed (Supplementary Data Fig. S3a-3, b-3, and c-3), in order to characterize the genetic relatedness among genotypes. The subpopulations identified in structure analyses were in line with the clustering pattern of the PCA. Moreover, the subpopulation containing well-known landraces (colored in yellow in the Fig. S3), appeared as substantially distant from the other subpopulations, when using SNPs from ‘Satonishiki’ and ‘PLov2-2n’ genomes in particular.

**Table 2:** Summary of SNP–trait associations related to sweet cherry fruit quality.

**Traits/reference genome**	**Chromosome**	**Physical position (bp)**	**Significance (*P* value)**	**Model**	***R*** ^**2a**^	**Allele** ^**b**^ **/effect** ^**c**^
**Fruit stem end cracking (SE)**						
Sweet cherry ‘Regina’	7	21 757 494	1.13e−10	MLMM	5.9	A/G
	7	26 772 180	4.88e−07	FarmCPU	3.9	A/C (0.01)
Sweet cherry ‘Satonishiki’	4	*4 381 656*	1.13e−10	MLMM	30.9	A/G
	1	26 464 729	2.00e−07	FarmCPU	20.9	C/A (0.01)
	4	*4 381 656*	1.38e−14		30.9	A/G (−0.07)
	7	17 821 793	2.98e−07		9.6	C/G (0.008)
	8	6 129 863	4.13e−07		16.0	C/T (0.01)
Peach ‘PLov2-2n’	1	18 149 428	1.13e−10	MLMM	4.8	A/T
	1	29 966 902	2.12e−09	FarmCPU	5.5	A/G (0.009)
	8	11 366 089	4.82e−09		6.0	G/C (0.03)
**Fruit pistillar end cracking**						
Sweet cherry ‘Regina’	4	6 606 690	1.38e−09	MLMM	8.9	T/C
	1	759 551	1.64e−09	FarmCPU	11.4	A/G (−0.01)
	6	22 196 234	6.48e−09		7.7	A/G (−0.009)
	6	31 626 357	1.10e−07		3.2	G/A (−0.02)
	7	16 540 822	1.77e−07		<0.1	C/T (0.007)
	7	24 643 127	6.15e−09		6.9	A/G (0.01)
Peach ‘PLov2-2n’	1	38 293 536	2.40e−09	MLMM	0.6	A/C
	4	*13 790 367*	9.98e−15		9.0	T/A
	1	13 745 047	1.74e−07	FarmCPU	2.8	T/C (−0.01)
	4	*13 790 367*	6.80e−16		9.0	T/A (−0.06)
	5	1 152 140	1.39e−08		7.4	A/G (−0.02)
	7	7 874 904	7.49e−11		4.9	A/G (−0.03)
**Fruit weight (FW)**						
Sweet cherry ‘Regina’	1	11 561 777	4.11e−13	FarmCPU	5.1	T/G (0.82)
	1	38 707 331	1.08e−09		2.5	A/T (0.44)
	1	**50 388 238**	5.52e−10		18.8	G/A (−0.57)
	2	**4 882 204**	7.96e−08		5.8	A/G (−0.31)
	3	15 787 719	1.34e−07		2.5	T/C (0.45)
	3	26 866 585	4.78e−08		1.6	T/C (−0.47)
	4	12 024 925	2.12e−07		17.2	A/G (−0.42)
	4	21 277 766	4.42e−09		1.9	A/G (0.54)
	6	11 112 467	1.59e−08		<0.1	T/C (0.51)
Peach ‘PLov2-2n’	2	28 325 332	8.87e−08	FarmCPU	7.3	G/A (0.36)
	3	**21 433 100**	6.06e−09		<0.1	A/C (−0.45)
**Fruit width**						
Sweet cherry ‘Regina’	1	**50 388 238**	8.60e−08	FarmCPU	15.0	G/A (−0.79)
	3	27 845 320	2.41e−09		<0.1	A/T (−0.61)
	7	**18 276 189**	4.17e−11		8.0	G/A (−0.70)
Peach ‘PLov2-2n’	1	8 612 931	1.48e−07	FarmCPU	<0.1	C/T (−0.69)
**Fruit thickness**						
Sweet cherry ‘Regina’	1	**50 388 238**	3.38e−10	FarmCPU	17.3	G/A (−1.00)
	2	**4 882 204**	3.23e−10		7.2	A/G (−0.68)
	2	7 007 862	4.33e−11		<0.1	A/C (−1.00)
	6	23 191 731	2.25e−07		3.1	T/C (0.79)
	7	**18 276 189**	2.98e−08		7.5	G/A (−0.60)
	7	22 298 828	1.51e−07		<0.1	A/C (−0.58)
Peach ‘PLov2-2n’	1	34 857 341	1.13e−07	FarmCPU	6.9	C/T (0.68)
	2	9 537 634	1.06e−10		8.6	T/C (0.82)
	3	**21 433 100**	3.25e−09		<0.1	A/C (−0.60)
	4	20 079 115	3.96e−07		12.6	A/G (0.45)
**Stone width**						
Sweet cherry ‘Regina’	2	2 834 950	6.79e−09	FarmCPU	18.8	T/G (0.20)
	3	1 281 929	1.32e−08		14.3	T/C (0.21)
	4	9 562 013	9.05e−09		11.1	T/C (−0.18)
	6	3 901 121	6.49e−10		7.4	T/C (0.28)
Peach ‘PLov2-2n’	3	4 481 012	1.40e−08	FarmCPU	13.3	C/T (−0.30)
	4	4 708 404	4.81e−09		0.4	A/G (−0.22)
	4	11 571 511	1.36e−07		1.2	A/C (−0.16)
**Stone thickness**						
Sweet cherry ‘Regina’	8	21 099 455	1.75e−07	FarmCPU	4.4	A/C (−0.25)
Sweet cherry ‘Satonishiki’	2	13 099 549	7.32e−15	FarmCPU	27.3	A/G (−0.41)
	3	12 470 834	3.69e−07		10.9	T/C (0.16)
	4	11 934 189	4.51e−10		14.8	T/A (−0.19)
	4	17 172 922	1.35e−07		3.9	T/A (−0.23)
	7	10 879 301	4.57e−07		<0.1	C/T (0.14)
Peach ‘PLov2-2n’	5	986 062	8.36e−08	FarmCPU	6.3	A/G (−0.37)
	8	12 886 531	2.10e−07		12.2	C/T (0.18)
**Stone shape in central view**						
Peach ‘PLov2-2n’	2	11 962 528	5.52e−12	FarmCPU	19.7	G/C (0.08)
	3	20 497 027	8.12e−09		3.3	A/C (−0.10)
	3	23 321 947	3.98e−13		1.8	C/G (−0.10)

Allelic effect not available for the MLMM model.

Physical positions in italics indicate identical marker–trait associations between GWAS models.

Physical positions in bold indicate identical marker–trait associations between traits.

a *R* ^2^ is the percentage of variance explained corrected for genome-wide background.

b The first allele mentioned is the minor allele.

c The allelic effect is the difference in mean of BLUPs between genotypes with one or other allele (sign: minor allele).

The LD estimates (measured as *r^2^*) and extent of LD decay were calculated using SNPs including those for which MAF was <5% (Supplementary Data Table S1). The genome-wide LD decayed, at 50 kb, to *r^2^* ~ 0.11 using the ‘Regina’ genome (Supplementary Data Fig. S4a), *r^2^* ~ 0.08 using the ‘Satonishiki’ genome (Supplementary Data Fig. S4-b) and *r^2^* ~ 0.03 using the ‘PLov2-2n’ genome (Supplementary Data Fig. S4c). As a consequence, we observed a faster LD decay when alignment of reads was performed on the *P. persica* genome compared with the two *P. avium* genomes.

### SNP–trait associations for fruit size and fruit-cracking-related traits

We compared a multilocus mixed model (MLMM) and the Fixed and random model Circulating Probability Unification (FarmCPU) method for all analyses. Using the ‘model selection’ option implemented in GAPIT, both kinship matrix and structure (principal component, PC) were included for all traits. However, according to the Bayesian information criterion (BIC), the best number of PCs to include for FW, for example, was zero (Supplementary Data Table S2). We checked whether the structure was influencing the 23 traits. We performed a PCA on the 23-BLUP (best linear unbiased prediction) phenotypic dataset, and we colored the individuals by their corresponding structure group (Supplementary Data Fig. S5). By obtaining overlapping ellipses of structure groups in the PCA of the traits, we observed no clear pattern of correlation between the phenotypes and the structure.

A total of 65 unique SNP–trait associations were found for eight traits, depending on the reference genome and the GWAS model (Table 2). We found SNPs significantly associated with the variation of fruit-cracking-related traits, fruit size (FW, FWi, and FT), stone size (SW and ST), and stone shape (SS). We did not find any SNPs associated with 15 traits, such as titratable acidity (TA), fruit side cracking (FS), fruit skin color (FC) and FJC.

**Figure 2:**
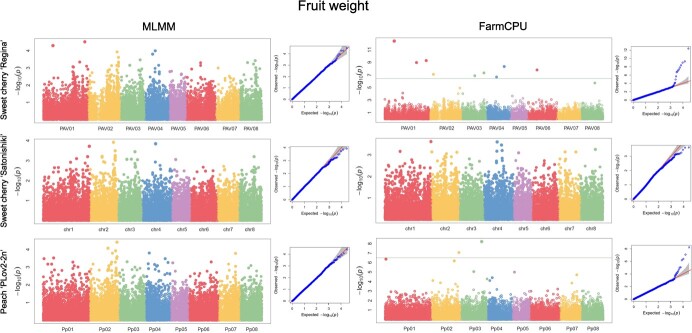
GWAS results for fruit weight. Manhattan plots followed by Q–Q plots using the MLMM (left) and FarmCPU (right) models for the three reference genomes. The horizontal line corresponds to the 1% Bonferroni threshold automatically implemented in GAPIT.

We found the highest number of associated SNPs with FW (Table 2). We obtained associated SNPs only considering the FarmCPU model, but we identified strong similar ‘hotspots’ in the Manhattan plots when comparing both models and the three reference genomes (Fig. 2). For instance, the two SNPs associated with FW at the beginning and end of chromosome 1 (11 561 777 and 50 388 238 bp) considering FarmCPU/‘Regina’ were also detected using MLMM, but were not significant. Moreover, the SNP associated in the middle of the chromosome 4 (12 024 925 bp) was detected in both models for ‘Regina’ and ‘Satonishiki’, but only significantly when considering FarmCPU/‘Regina’. Similarly, the hotspot at the end of the chromosome 2 was found in all configurations, but was only significant when considering FarmCPU/‘PLov2-2n’.

**Figure 3:**
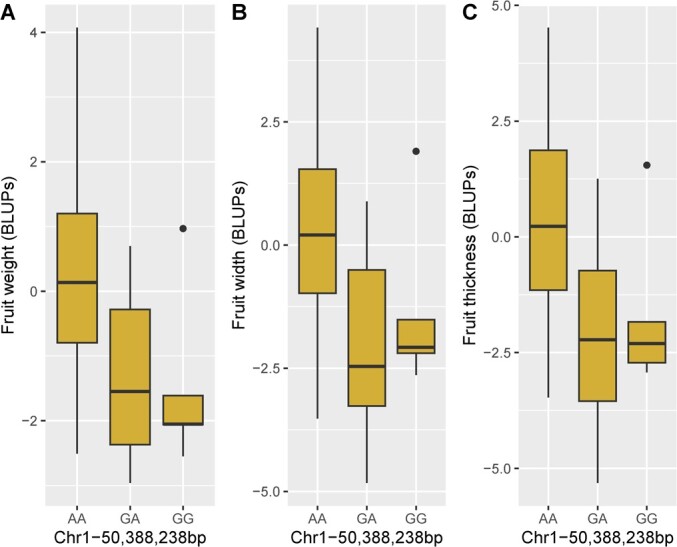
Boxplots of the allele effects for the SNP Chr1-50 388 238 bp associated with global fruit size using FarmCPU/’Regina’. **A** Fruit weight **B** Fruit width. **C** Fruit thickness.

The SNP Chr1-50 388 238 bp, detected considering FarmCPU/‘Regina’, explained the largest part of FW variance (18.8%), with the major allele A as favorable (vs allele G with an estimated effect of −0.57 on the corresponding BLUP value). This SNP was also significantly associated with two correlated traits: FWi (15.0% of the variance explained) and FT (17.3%). The boxplots of the alleles of this SNP, for the three traits, are shown in Fig. 3. In addition, the SNP Chr2-4 882 204 bp, detected considering FarmCPU/‘Regina’, explained 5.8 and 7.2% of FW and FT variances, respectively, with the major allele G as favorable (vs allele A with an estimated effect of −0.31 and −0.68 on the corresponding BLUP values, respectively). Similarly, the SNP Chr7-18 276 189 bp, detected considering FarmCPU/‘Regina’, was associated with FWi and FT. Altogether, these three SNPs explained 32.0% of FT variance (Table 2).

We identified another association with global fruit size considering FarmCPU/‘PLov2-2n’. The SNP Chr3-21 433 100 bp was associated with FW and FT, but explained <0.1% of the variance for both traits. For FW, we detected another associated SNP considering FarmCPU/‘PLov2-2n’ and the SNP Chr2-28 325 332 bp, explaining 7.3% of the variance. We did not find associations for any of these three fruit size-related traits using ‘Satonishiki’.

Finally, concerning fruit-cracking-related traits, we detected associations for both fruit stem end cracking (SE) and fruit pistillar end cracking (PI) (Table 2). As previously observed for fruit size-related traits, we found close hotspots in the Manhattan plots when comparing the different models and genomes, but with different significant SNP–trait associations (Supplementary Data Fig. S6). All together, we found 9 associated loci on chromosomes 1, 4, 7, and 8 for SE and 11 associations on chromosomes 1, 4, 5, 6, and 7 for PI (Table 2; Supplementary Data Fig. S6). The associated SNP Chr4-4 381 656 bp was significant in both GWAS models and explained the largest effect (30.9%) for SE. Considering FarmCPU/‘Satonishiki’, the total SE variance explained by the three best SNPs reached 67.8%.

### SNP–trait associations for stone-related traits

For stone-size-related traits, we found seven and eight SNP–trait associations for SWi and ST, respectively. Contrary to fruit-size-related traits, we did not find any locus in common between these two highly correlated traits (Table 2), while we observed similar hotspots between both traits (Supplementary Data Fig. S7). For example, we identified two significant loci at the beginning of chromosomes 3 and 4 associated with SWi considering FarmCPU/‘Regina’ that corresponded to non-significant signals for ST. The associated SNPs that explained the largest part of the SWi and ST variances were both located on chromosome 2 (Chr2-2 834 950 bp, *R*^2^ 18.8%, FarmCPU/‘Regina’ for SWi, and Chr2-13 099 549 bp, *R*^2^ 27.3%, FarmCPU/‘Satonishiki’ for ST). For SS, the associated SNP that explained the largest part of the variance was also on chromosome 2, considering FarmCPU/‘PLov2-2n’ (Chr2-11 962 528 bp, *R*^2^ 19.7%).

**Figure 4:**
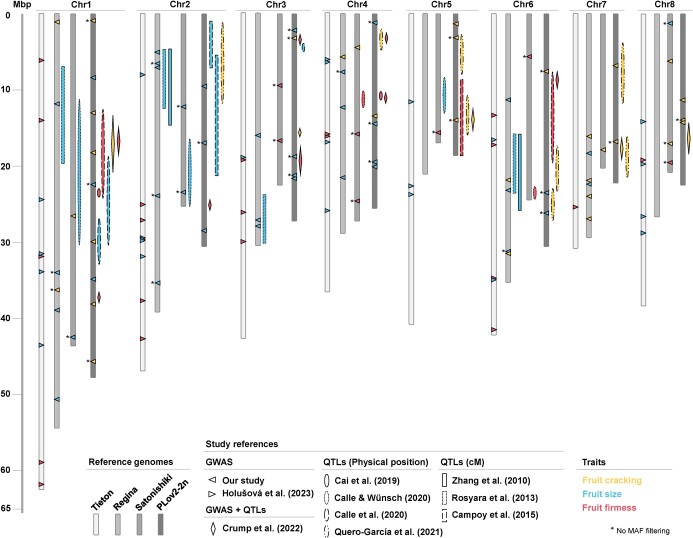
Global representation of the genetic architecture of fruit quality traits in sweet cherry. Each marker–trait association shape correspond to a study: Holušová *et al*. [26], GWAS using SNPs mapped on ‘Tieton’; Crump *et al*. [14], GWAS and QTL mapping using SNPs mapped on ‘PLov2-2n’; Cai *et al*. [10], QTL mapping on multiple progenies and SNPs mapped on ‘PLov2-2n’; [13], QTL mapping on multiple progenies and SNPs mapped on ‘Satonishiki’ (major loci); Calle *et al*. [12], QTLs on one progeny and SNPs mapped on ‘PLov2-2n’; Zhang *et al*. [17], QTLs on one progeny and SSRs coming from *Prunus* species (QTLs in centimorgan placed on ‘Regina’); Rosyara *et al*. [16], QTLs on multiple progenies and SSRs and SNPs coming from *Prunus* species (QTLs with intervals given in centimorgan placed on ‘Regina’); Campoy *et al*. [11], QTLs on multiple progenies and SNPs mapped on ‘PLov2-2n’ (QTLs with intervals given in centimorgan and major loci); Quero-García *et al*. [18], QTLs on multiple progenies and SNPs mapped on ‘PLov2-2n’ (QTLs with intervals given in centimorgan and major loci). The QTLs are placed to the right of the corresponding chromosome. We divided all traits into three main categories, colored in the figures as follows: cracking-related traits in yellow, global fruit-size-related traits in blue, and firmness-related traits in red.

### Comparison of GWAS results including or not minor allele frequency filtering

When we compared the GWAS results including or not including MAF filtering, we found associations for the same traits (SE, FW, FWi, FT, SWi, and ST), and we also identified SNPs associated with fruit firmness (FF) (Supplementary Data Table S3). For each trait, only one or two SNP–trait associations were strictly identical or at a highly close position to those previously identified. For instance, for SE we identified 10 and 11 associated SNPs, all GWAS models and genomes combined, including and not including MAF filtering, respectively, but only the SNP Chr1-26 464 729 bp was found in both lists, considering FarmCPU/‘Satonishiki’.

However, as was the case when comparing GWAS models, the Manhattan plots showed similar strong hotspots when we compared the results including or not including MAF filtering. For example, for FW considering the ‘Regina’ genome, we observed in both cases hotspots in chromosomes 1, 2, 3, 4, and 6, using the MLMM model (Supplementary Data Fig. S8).

Concerning FF, we identified seven associated SNPs using the ‘Satonishiki’ genome (Supplementary Data Table S3), while we did not obtain any significant SNP when we included MAF filtering. Using the MLMM model, we found the SNP Chr4-15 932 813 bp, which explained 20.6% of the variance, with an allelic effect of 10.7 for the favorable A major allele. Using the FarmCPU model, we identified additional loci on chromosomes 3, 4, 5, 6, and 8.

### Colocalizations of SNP–trait associations identified between different reference genomes

In order to identify potentially colocalizing SNP–trait associations of fruit quality traits in sweet cherry, we summarize results from this study and nine previously published articles (Fig. 4). Interestingly, we found an SNP on chromosome 4 at 15.93 Mbp using ‘Satonishiki’ for FF (no MAF filtering results in Supplementary Data Table S3), where Crump *et al*. [14] identified an SNP associated with the same trait using the ‘Tieton’ genome at 16.00 Mbp. We searched for colocalizations of close SNPs associated with the same trait (or strongly correlated traits) between two genomes by using BLAST with the surrounding sequence (Supplementary Data Table S4). For FF, the two positions previously cited on chromosome 4 are not so close since the sequence around 15.93 Mbp of ‘Satonishiki’ corresponded to a sequence around 17.99 Mbp on ‘Tieton’. Consequently, these two loci, thought at first to be colocalizing, are actually more physically distant. This was the case for the largest part of the SNP–trait associations potentially colocalizing that we focused on, particularly for FF and fruit size-related traits (Supplementary Data Table S4). However, although they did not perfectly colocalize, the SNP–trait associations for fruit-cracking-related traits can be considered as physically close. We also identified three colocalizations, including an SNP for SE on chromosome 4 at 4.38 Mbp using ‘Satonishiki’, corresponding to a locus at 3.46 Mbp on ‘PLov2-2n’,which colocalized with a QTL identified by Quero-García *et al*. [18] using the ‘PLov2-2n’ genome between 1.98 and 3.92 Mbp. Interestingly, if the potentially colocalizing loci are physically distant, the framing genes have in most cases the same annotation (Supplementary Data Table S4).

### Identification of genes putatively involved in fruit quality

Based on the association results, we searched for candidate genes and their predicted functions in the upstream and downstream flanking regions of all significant SNP–trait associations, defined by the LD blocks and considering MAF filtering. The size of LD block intervals varied significantly, with an average of 20 772 bp (from no block identified to a block of 134 216 bp). First, we explored associated SNPs within gene coding sequences, here referred to as ‘putatively best candidate genes’ (Table 3).

**Table 3:** Summary of LD block intervals and putatively best candidate genes.

**Trait/reference genome**	**Chromosome**	**Physical position (bp)**	**Model**	**LD block interval (bp)** ^**a**^	**Gene ID**	**Gene interval**	**TAIR description**	**InterPro description**	**Mercator description**
**Fruit stem end cracking (SE)**									
Sweet cherry ‘Regina’	7	21 757 494	MLMM	21 712 538–21 774 033	PAV07_REGINAg0341131	21 756 525–21 758 643	Cyclin-dependent kinase inhibitor family protein		Cell cycle organization, cell cycle control, cyclin-dependent regulation, inhibitor (KRP/ICK)
	7	26 772 180	FarmCPU	26 772 152–26 840 041	PAV07_REGINAg0352621	26 772 111–26 775 310	α/β-Hydrolases superfamily protein		Not assigned
Sweet cherry ‘Satonishiki’	4	*4 381 656*	MLMM	4 381 635–4 381 656	Pav_sc0001289.1_g020.1.mk	4 376 343–4 382 973		MADS-box transcription factor	
	1	26 464 729	FarmCPU	26 462 653–26 501 227	Pav_sc0000893.1_g1130.1.mk	26 460 477–26 467 549		Proton-dependent oligopeptide transporter family	
	4	*4 381 656*		4 381 635–4 381 656	Pav_sc0001289.1_g020.1.mk	4 376 343–4 382 973		MADS-box transcription factor	
	7	17 821 793		17 814 961–17 831 793	No gene				
	8	6 129 863		6 129 845–6 129 864	No gene				
Peach ‘PLov2-2n’	1	18 149 428	MLMM	No block	Prupe.1G193500	18 146 524–18 155 251	RNA helicase family protein		
	1	29 966 902	FarmCPU	29 952 789–29 966 902	No gene				
	8	11 366 089		11 328 691–11 366 089	Prupe.8G080100	11 365 948–11 367 159	SAUR-like auxin-responsive protein family		
**Fruit pistillar end cracking**									
Sweet cherry ‘Regina’	4	6 606 690	MLMM	6 552 892–6 671 623	No gene				
	1	759.551	FarmCPU	757 895–759 563	PAV01_REGINAg0001161	758 350–762 796	HMG (high mobility group) box protein with ARID/BRIGHT DNA-binding domain		
	6	22 196 234		22 124 957–22 201 100	PAV06_REGINAg0292311	22 193 385–22 196 564	Nucleotide-sugar transporter family protein		Solute transport, carrier-mediated transport, DMT superfamily, NST-TPT group, nucleotide sugar transporter (ROCK)
	6	31 626 357		No block	PAV06_REGINAg0309481	31 625 130–31 627 159	Pectin lyase-like superfamily protein		Cell wall organisation, pectin, homogalacturonan, modification and degradation, pectin methylesterase
	7	16 540 822		16 540 367–16 540 822	PAV07_REGINAg0332031	16 539 830–16 543 136	DNA-binding bromodomain-containing protein		RNA biosynthesis, transcriptional regulation, MYB transcription factor superfamily, transcription factor (MYB-related)
	7	24 643 127		24 635 983–24 682 873	PAV07_REGINAg0347731	24 640 962–24 643 592	Not assigned		Not assigned
Peach ‘PLov2-2n’	1	38 293 536	MLMM	38 281 173–38 293 553	No gene				
	4	*13 790 367*		13 788 760–13 790 367	Prupe.4G219300	13 789 348–13 791 624	RTL2, ATRTL2		RTL2 RNAse THREE-like protein 2
	1	13 745 047		No block	No gene				
	4	*13 790 367*	FarmCPU	13 788 760–13 790 367	Prupe.4G219300	13 789 348–13 791 624	RTL2, ATRTL2		
	5	1 152 140		1 152 140–1 155 059	Prupe.5G011100	1 135 738–1 152 387	Patched family protein		
	7	7 874 904		7 874 878–7 881 483	No gene				
**Fruit weight**									
Sweet cherry ‘Regina’	1	11 561 777	FarmCPU	11 561 777–11 634 064	PAV01_REGINAg0018861	11 561 343–11 562 826	Soybean gene regulated by cold-2, SRC2		
	1	38 707 331		38 706 836–38 707 331	PAV01_REGINAg0053471	38 707 066–38 710 860	**2-Oxoglutarate (2OG) and Fe(II)-dependent oxygenase superfamily protein**		Protein modification, hydroxylation, prolyl hydroxylase
	1	**50 388 238**		No block	PAV01_REGINAg0076531	50 388 016–50 388 741	Not assigned		Not assigned
	2	**4 882 204**		No block	No gene				
	3	15 787 719		15 714 103–15 846 318	No gene				
	3	26 866 585		26 866 585–26 866 593	PAV03_REGINAg0175791	26 866 029–26 869 380	ABI five binding protein 3		RNA biosynthesis, transcriptional regulation, transcriptional repression, transcriptional co-repressor (AFP/NINJA)
	4	12 024 925		11 991 001–12 090 994	No gene				
	4	21 277 766		21 277 766–21 277 974	PAV04_REGINAg0221611	21 277 132–21 279 320	Glycosyl hydrolase superfamily protein, glucan endo-1,3-beta-glucosidase		Enzyme classification, hydrolases, glycosylase
	6	11 112 467		10 978 251–11 112 467	PAV06_REGINAg0281641	11 109 723–11 113 261	Protein kinase superfamily protein		
Peach ‘PLov2-2n’	3	**21 433 100**	FarmCPU	21 422 088–21 433 100	No gene				
	2	28 325 332		28 303 994–28 328 333	Prupe.2G287700	28 324 854–28 329 981	**2-Oxoglutarate (2OG) and Fe(II)-dependent oxygenase superfamily protein**		
**Fruit width**									
Sweet cherry ‘Regina’	1	**50 388 238**	FarmCPU	No block	PAV01_REGINAg0076531	50 388 016–50 388 741	Not assigned		Not assigned
	3	27 845 320		27 845 320–27 845 442	PAV03_REGINAg0178001	27 843 561–27 846 383	3-dehydroquinate synthase		Not assigned
	7	**18 276 189**		18 276 189–18 304 211	PAV07_REGINAg0334531	18 273 442–18 276 816	Fasciclin-like arabinogalactan protein 17 precursor		Cell wall organisation, cell wall proteins, arabinogalactan-protein (AGP) activities, fasciclin-type AGP (FLA)
Peach ‘PLov2-2n’	1	8 612 931	FarmCPU	8 612 877–8 612 931	No gene				
**Fruit thickness**									
Sweet cherry ‘Regina’	1	**50 388 238**	FarmCPU	No block	PAV01_REGINAg0076531	50 388 016–50 388 741	Not assigned		Not assigned
	2	**4 882 204**		No block	No gene				
	2	7 007 862		7 007 862–7 023 349	PAV02_REGINAg0091811	7 006 930–7 007 892	Terpenoid synthases superfamily protein		Not assigned
	6	23 191 731		23 191 731–23 191 802	No gene				
	7	**18 276 189**		18 276 189–18 304 211	PAV07_REGINAg0334531	18 273 442–18 276 816	Fasciclin-like arabinogalactan protein 17 precursor		Cell wall organisation, cell wall proteins, arabinogalactan-protein (AGP) activities, fasciclin-type AGP (FLA)
	7	22 298 828		No block	PAV07_REGINAg0342301	22 295 799–22 299 256	Zinc-finger domain of monoamine-oxidase A repressor R1		
Peach ‘PLov2-2n’	1	34 857 341	FarmCPU	34 844 765–34 857 341	Prupe.1G391000	34 856 718–34 859 237	PPPDE putative thiol peptidase family protein		
	2	9 537 634		No block	No gene				
	3	**21 433 100**		21 422 088–21 433 100	No gene				
	4	20 079 115		20 079 098–20 079 115	No gene				
**Stone width**									
Sweet cherry ‘Regina’	2	2 834 950	FarmCPU	2 826 953–2 834 950	PAV02_REGINAg0087361	2 834 698–2 861 437	Nuclear RNA polymerase C2		RNA biosynthesis, DNA-dependent RNA polymerase (Pol) complexes, subunit 2 of Pol III RNA polymerase
	3	1 281 929		1 281 926–1 281 932	PAV03_REGINAg0140531	1 280 853–1 282 913	SGNH hydrolase-type esterase superfamily protein		Not assigned
	4	9 562 013		9 561 952–9 562 332	PAV04_REGINAg0202751	9 560 636–9 563 662	Lectin protein kinase family protein		Protein modification, TKL protein kinase superfamily, G-Lectin protein kinase families, protein kinase (SD-1)
	6	3 901 121		No block	PAV06_REGINAg0270161	3 900 837–3 901 639	Protein kinase superfamily protein		Not assigned
Peach ‘PLov2-2n’	3	4 481 012	FarmCPU	4 454 894–4 543 647	Prupe.3G062600	4 475 112–4 484 075	Calmodulin-interacting protein 111		
	4	4 708 404		4 703 492–4 720 613	Prupe.4G093400	4 705 881–4 708 801	Cell wall-associated kinase		
	4	11 571 511		11 571 511–11 571 518	Prupe.4G192200	11 563 411–11 571 968	Ubiquitin-specific protease 14		
**Stone thickness**									
Sweet cherry ‘Regina’	8	21 099 455	FarmCPU	21 099 422–21 099 480	PAV08_REGINAg0389731	21 098 040–21 100 558	acyl-CoA oxidase 5, cytochrome P450 87A3		Enzyme classification, oxidoreductases, oxidoreductase with incorporation or reduction of molecular oxygen
Sweet cherry ‘Satonishiki’	2	13 099 549	FarmCPU	13 099 530–13 099 549	No gene				
	3	12 470 834		12 469 296–12 471 534	No gene				
	4	11 934 189		11 930 389–11 934 189	Pav_sc0000584.1_g720.1.mk	11 933 227–11 934 268		Seipin family	
	4	17 172 922		17 172 408–17 172 922	No gene				
	7	10 879 301		10 879 299–10 879 341	Pav_sc0000036.1_g140.1.br	10 877 394–10 879 784		Reverse transcriptase zinc-binding domain	
Peach ‘PLov2-2n’	5	986.062	FarmCPU	No block	Prupe.5G009400	973 835–986 555	RAD3-like DNA-binding helicase protein		
	8	12 886 531		12 879 439–12 887 734	Prupe.8G096600	12 882 693–12 887 019	Mitochondrial transcription termination factor family protein		
**Stone shape in central view**									
Peach ‘PLov2-2n’	2	11 962 528	FarmCPU	11 962 521–11 962 545	No gene				
	3	20 497 027		No block	Prupe.3G193300	20 495 590–20 497 376	Not assigned		
	3	23 321 947		No block	No gene				

Physical positions in italics indicate identical marker–trait associations between GWAS models.

Physical positions in bold indicate identical marker–trait associations between traits.

Candidate genes in bold are identical.

a LD block intervals determined using Solid spine of LD method implemented in Haploview software.

For the traits related to fruit size (FW, FWi, and FT), we identified 11 candidate genes, including two genes encoding a 2-oxoglutarate and Fe(II)-dependent oxygenase protein (PAV01_REGINAg0053471 on chromosome 1 of ‘Regina’ and Prupe.2G287700 on chromosome 2 of ‘PLov2-2n’) and genes encoding a glucan endo-1,3-β-glucosidase, a terpenoid synthase, and a fasciclin-like arabinogalactan protein 17 precursor.

Regarding fruit-cracking-related traits, we identified 12 candidate genes: six for SE, such as a cyclin-dependent kinase inhibitor protein, a MADS-box transcription factor, and a SAUR-like auxin-responsive protein, and six for PI , which encoded e.g. a nucleotide–sugar transporter protein, a pectin lyase-like protein, and an RTL2 RNAse THREE-like protein. We finally pinpointed 12 additional candidate genes for stone-size-related traits, such as three protein kinases, including a cell-wall-associated kinase.

More globally, we also considered additional candidate genes, corresponding to all genes identified within the defined LD blocks for the SNP–trait association (Supplementary Data Table S5). For a given trait, we identified some candidate genes on different chromosomes but with identical or similar functional annotation using the different reference genomes. For instance, for SE we found genes encoding calcium-dependent protein kinase and putative calcium-transporting ATPase using ‘Regina’, while a gene encoding a probable calcium-binding protein was identified using the ‘PLov2-2n’ genome. Additionally, for SE we observed genes encoding different types of glycosyltransferases using the three genomes (putative xylogalacturonan β-1,3-xylosyltransferase for ‘Regina’, UDP-glucosyltransferase for ‘Satonishiki’, and probable galacturonosyltransferase-like for ‘PLov2-2n’).

Concerning FW, we found genes encoding various transporters (MATE, sugar, and solute), transcription factors (MYB, Hap3/NF-YB, and DOG), and RLK/Pelle kinases (for four of the nine associated LD blocks) using ‘Regina’. Several candidate genes were specific for traits FWi and FT, such as genes encoding a fasciclin-like arabinogalactan protein and a terpenoid synthase, and we found genes encoding ethylene response proteins potentially involved in global fruit size variation (putative ethylene-responsive binding factor-associated repression ninja family for FW using ‘Regina’ and ethylene-responsive transcription factor for FT using ‘PLov2-2n’).

Finally, we found genes encoding a putative LRR receptor-like serine/threonine protein kinase potentially involved in ST and stone shape using ‘Satonishiki’ and ‘PLov2-2n’ genomes, and F-box domain protein-encoding genes using the ‘PLov2-2n’ genome for the same traits. Then, for global stone size (SWi and ST) we found genes previously identified for fruit-related traits, such as genes encoding a RLK/Pelle kinase, a GDSL lipase, and an AP2 transcription factor.

## Discussion

### Contrasted heritabilities are associated with categories of traits

Large phenotypic variability was found for most traits, and was comparable to that found in previously published GWAS on sweet cherry. For instance, the range of FH values, from 12.76 to 24.34 mm (average 18.73 mm), was comparable to those obtained for fruit length by Holušová *et al*. [26], from 16.70 to 26.98 mm (average 21.31 mm). Regarding FW, our values ranged from 1.70 to 16.61 g (average 5.68 g), while Holušová *et al*. [26] obtained a smaller range, from 2.88 to 10.98 g (average 5.97 g), proving the large variability of the phenotypes of our germplasm collection.

Some traits could be considered as normally distributed, i.e. suitable to be efficiently exploited in GWAS studies. For example, FF was normally distributed, as observed in Crump *et al*. [14]. However, a few distributions, such as for cracking-related traits, were found to be skewed. Between 2014 and 2019, our germplasm collection location experienced climatic conditions with low values of rainfall, resulting in a large number of genotypes with zero cracked fruits. Moreover, the variable transformation of these phenotypes (square root of the arcsine) did not improve their distributions. Left-skewed distributions were already observed for cracking-related traits by Quero-García *et al*. [18], whereas Crump *et al*. [14] showed a right-skewed distribution of cracking incidence in 2020. This was likely due to the increased total rainfall and the less accurate fruit cracking assessment method performed by Crump *et al*. [14]. However, no distribution showed a multimodal pattern, except for FC, FJC, and FPE. Similar asymmetric phenotypic distributions were reported in other fruit species, such as apple [39].

We found that FW and fruit-size-related traits were positively correlated (from 0.88 to 0.98), as reported in the work of Holušová *et al*. [26], where FW was positively correlated with fruit length (0.90), FWi (0.94), and FT (0.93). In our study, FW tended to be slightly positively correlated with FF (0.45), as already noted in sweet cherry by Piaskowski *et al*. [40], with a positive correlation of 0.51. However, Campoy *et al*. [11] found negative correlations using two mapping progenies, probably due to the unique genetic background of the three parents involved (‘Regina’, ‘Lapins’, and ‘Garnet’). The correlation between FW and FF shows inconstant behavior within Rosaceae: the correlation was found negative in apple [41], close to zero in strawberry [42], and positive in peach [43]. Moreover, a negative correlation between TA and fruit size was reported by Piaskowski *et al*. [40], while we found a negative correlation between pH and fruit size, juice pH and TA being negatively correlated.

Globally, besides the low values observed for a few traits, heritabilities were moderate to high, which is indicative of good reproducibility of the phenotyping and a relevant genetic contribution to the observed variability in the measured traits, thereby offering an excellent opportunity for improving these traits through selection. Our calculated broad-sense heritabilities showed high values for traits related to fruit and stone size, as observed in previous studies on sweet cherry [11, 15–17, 40]. Furthermore, our results were also consistent with previous work, which reported high values of *H*^2^ for FF (*H*^2^ = 0.73–0.97 or *h*^2^ = 0.70) in sweet cherry [10, 11, 14, 40].

For fruit cracking traits, our findings demonstrated a moderate to low broad-sense heritability (*H*^2^ values ranged from 0.36 for FS to 0.64 for PI). A similar trend was found by Quero-García *et al*. [18] using mapping progenies, with a higher value for PI, and our *H*^2^ values were also in line with the work of Crump *et al*. [14], which obtained *h*^2^ = 0.54 for cracking incidence. Nevertheless, the values estimated in our study for such traits were higher compared with the ones obtained in other fruit species, such as apple where *H*^2^ = 0.03–0.22 [39, 44]. Low heritability values are known for this category of traits that are strongly influenced by the environment [7]. For the traits concerned, the lower values of heritability might also be partially explained by the availability of only two years of data.

### Phenotypic variances are not affected by sweet cherry population structure

The two approaches used to analyze the structure of the sweet cherry panel provided complementary information that was in line with previous results obtained by Campoy *et al*. [45] in the INRAE sweet cherry germplasm collection (210 individuals) using the RosBREED 6 *K* array. These authors first identified two groups, mainly constituted by bred cultivars on the one hand and landraces on the other hand. Our analyses confirmed this first structure since the distinct group obtained using SNPs from the ‘PLov2-2n’ genome contained exclusively landraces and mainly the same as those identified by Campoy *et al*. [45]. Regarding the substructure, as in our case, Campoy *et al*. [45] and Barreneche *et al*. [46] both identified a subgroup constituted by particular interesting French landraces, such as ‘Saint Georges’, ‘Xapata’, and ‘Durette’. The choice of the best *K* value (number of inferred subpopulations or clusters within the dataset) can be influenced by the reference genome considered to obtain the set of SNPs, because the reference genome is used to align and identify genetic variations in the sample genomes. Different reference genomes may have variations in the regions they cover, leading to the identification of different SNPs (and different missing data) and differences in the genetic diversity captured by the SNPs, and potentially influencing the inferred structure.

We checked whether the structure was influencing the phenotypic variance of the 23 traits and we observed no clear pattern of correlation between the phenotypes and the structure. As a consequence, all the traits did not seem to be correlated with the structure. The influence of population structure on phenotypic variance was weak, maybe because kinship accounted partially for the structure. This was observed in other studies using GAPIT in sweet cherry [14, 26] and in walnut [47], and explained why no PC was considered as a structure covariable in further GWAS analyses using the BIC.

For all reference genomes, the LD was estimated as *r^2^* and dropped below 0.2 within a distance of <50 kb. This value is in agreement with previous studies, where *r^2^* < 0.2 was found at ~100 kb in 210 sweet cherry accessions using 1215 SNPs not LD-pruned [45] or with 35 SSRs [48], and in Xanthopoulou *et al*. [49], where LD decayed to 90% at 55 kb using whole-genome re-sequencing. Differences in the number of markers used (>70 000 SNPs for the two sweet cherry genomes vs >160 000 SNPs for the *P. persica* genome) may affect the LD value, as reported in the literature, where LD estimates tended to be higher with denser SNP panels [50, 51]. In this study we used GBS, where the alignment accuracy of short-read sequences is affected by the choice of the reference genome [52], which would explain the differences obtained for the SNP sets, and consequently, the LD decay estimations. Globally, the fast LD decay in our sweet cherry population is helpful in the search for candidate genes, but requires a large number of SNPs. As a rough guide, with a sweet cherry genome size estimated at 270 Mb and *r*^2^ = 0.2 estimated at ~5 kb in our panel, we would need an SNP set of ~50 000 SNPs. With 30 000 SNPs on average in our study, we probably did not capture the entirety of loci potentially involved in the variation of a trait and the number of SNPs was lower than in Holušová *et al*. [26] (1 767 106 SNPs), but much higher than in Crump *et al*. [14] (3302 SNPs).

### SNP–trait associations are dependent on data dimension, GWAS model, reference genome and MAF filtering

In addition to structure and relatedness, our study highlights several other factors that influence significantly the loci associated with a trait: GWAS model, reference genome, and MAF filtering. Firstly, the number of accessions and the degree of their phenotypic variations can influence the reliability of GWAS analysis. The number of accessions used in this study may seem low in order to achieve statistical power [22, 53]. However, several studies in fruit species have recently detected significant SNP–trait associations with panels of <200 accessions: 172 genotypes in apple [54] and even 132 in peach [34]. In Crump *et al*. [14], 259 individuals were retained for sweet cherry GWAS analyses but they were seedlings from 22 breeding parents and may represent a lesser level of phenotypic variability. Our sampling could therefore be suitable for GWAS analyses, according to our *H*^2^ values and phenotypic ranges.

Moreover, although our study highlighted different results when comparing different models and different reference genomes, the patterns of hotspots were very close in our study and gave weight to our significant SNP–trait associations. In our study, the FarmCPU model identified more SNP–trait associations than the MLMM. The performance of FarmCPU was already highlighted in a soybean simulated dataset, as it consistently identified a single highly significant SNP close to known published genes for six qualitative traits [55]. Furthermore, we chose to work with three genomes because the role of the reference genome used for the mapping of short reads is crucial [56]. This is also in accordance with previous work in maize that proved the usefulness of genotyping datasets obtained from multiple reference genomes [57]. We used sweet cherry and peach genomes, which share a high level of synteny [58], and for years the *P. persica* genome was the *Prunus* reference genome. Our study showed that the set of SNPs retained after alignment and filtering were different between genomes.

Finally, considering MAF filtering or not can also be crucial. In our analysis, when we included SNPs with MAF <5%, and even if the original set of SNPs with MAF >5% was conserved, we had mostly different SNPs associated with traits (even on different chromosomes). In other words, the increase in the number of SNPs included in our analyses strongly influenced the GWAS significance. In Holušová *et al*. [26] and Crump *et al*. [14], MAF filtering was set to 1% but both used >200 accessions. Since in our study we may have had a limited number of individuals and a limited number of SNPs, we rather considered results including MAF filtering primarily. However, it could be interesting to focus on the SNP–trait associations obtained without MAF filtering, as proposed by Tabangin *et al*. [59].

### Complexity of the genetic architecture of fruit quality traits in sweet cherry

Our study highlighted the complex genetic determinism of fruit quality traits, which are highly quantitative. For instance, we identified 11 SNPs associated with FW at distinct loci using all GWAS models and genomes, and 6 additional SNPs, when considering SNPs with MAF <5%.

The global size of cherry fruit (including FW, FWi, FH, and FT) is a trait that researchers have particularly focused on. As in different QTL studies focusing on FW [11, 16], we found several SNPs associated with FW and its dimensions at distant positions within chromosome 2. A major locus was previously found at the beginning of chromosome 2 at 8.0 Mbp for FWi [26] and FW [11, 16, 17]. Our analyses confirmed this locus as we found five SNPs associated with FW, FWi, and/or FT using the three genomes within an interval between 4.9 and 12.0 Mbp at the beginning of chromosome 2.

We also confirmed another major locus identified at the end of the chromosome 2 for FW and size in previous studies [13, 26]. Our study highlighted six SNPs associated with FW, FWi, and/or FT using the three genomes within an interval between 23.1 and 36.5 Mbp at the end of chromosome 2. Moreover, when we considered the SNPs with MAF <5%, the major loci were those at the end of chromosome 2, as reported by Holušová *et al*. [26]. When applying MAF filtering, the locus explaining the largest percentage of FW variance was at the end of chromosome 1. As a consequence, considering no MAF filtering could be more conceivable when results are in line with those of previous published articles.

Additionally, we found several SNPs close to previously identified loci for these fruit size-related traits, particularly on chromosomes 1 and 6 (Fig. 4). Campoy *et al*. [11] found SNPs associated with FW on all chromosomes, except chromosomes 4 and 7, and Holušová *et al*. [26] found loci involved in FW and fruit size-related traits on all chromosomes, except chromosome 7. Our study showed the complex genetic determinism of global cherry size as we found associated SNPs on each chromosome, except chromosome 5. We found several identical SNPs involved in positively correlated fruit-size-related traits on chromosomes 1, 2, 3, and 7 when MAF filtering was applied (chromosomes 1, 2, 4, and 6 when MAF filtering was not applied), while Holušová *et al*. [26] identified similar SNPs also involved in positively correlated fruit-size-related traits on chromosomes 1, 2, 4, and 5. As a consequence, our results suggest that including correlated traits in GWAS can give clues for the identification of major loci shared by these traits, but can also identify loci more specific for one trait. This finding can be useful in post-GWAS prioritization, first focusing on very close loci associated with multiple correlated traits.

For FF, we identified associated SNPs only when including those with MAF <0.5. A major locus is mentioned in the literature at the beginning of chromosome 4 [10, 13, 14] but, surprisingly, we did not find any SNP–trait association at this locus. We also did not find an FF-associated locus on chromosome 1, yet one was mentioned in the literature [10, 11, 14, 26]. However, we identified one SNP using ‘Satonishiki’ on chromosome 4 at 15.9 Mbp explaining 20.6% of the variance, and Holušová *et al*. [26] mentioned that their locus explaining the largest part of the FF variance measured by hand (1, very soft; 9, very firm) was also on chromosome 4 at 16.0 Mbp using ‘Tieton’. In the same way, we identified an SNP using ‘Satonishiki’ on chromosome 8 at 19.6 Mbp, whereas Holušová *et al*. [26] found a significant SNP at 19.2 Mbp using ‘Tieton’, explaining the largest part of the FF variance measured by a texturometer.

Regarding cracking-related traits, we identified SNP–trait associations colocalizing with QTLs associated with PI [18]. We also found three loci colocalizing with QTLs reported by Quero-García *et al*. [18] using ‘PLov2-2n’, but for different cracking positions: (i) we identified an SNP associated with PI at 7.9 Mbp on chromosome 7, while Quero-García *et al*. [18] identified a QTL for FS between 4.3 and 12.1 Mbp; (ii) we identified an SNP associated with SE at 14.7 Mbp on chromosome 5, while Quero-García *et al*. [18] identified a QTL for PI between 10.7 and 15.5 Mbp; and (iii) we identified an SNP associated with PI at 17.7 Mbp on chromosome 7, while Quero-García *et al*. [18] identified a QTL for SE between 17.0 and 20.8 Mbp. Moreover, a promising QTL was also found controlling up to 20–25% of SE variance between 1.98 and 3.92 Mbp on chromosome 4 using ‘PLov2-2n’ [18], and we identified an SNP using ‘Satonishiki’ highly associated with this trait within the QTL interval. As expected, several loci related to cracking explained part of the phenotypic variance for PI and FS, or for SE and FS, since it has been proven that FS is mostly an extension of PI and SE [60]. On the other hand, PI and SE occur at opposite regions of the cherry fruit, but given the highly complex and polygenic nature of cracking tolerance, it is likely that loci controlling PI and SE may coexist in proximal genomic regions. Although the methodology for evaluating cracking tolerance in our study was the same as the one reported by Quero-García *et al*. [18], the nature of the studied material and their location and observation periods clearly differed. In particular, high levels of rainfall at harvest during several study years were reported for the first study [18], while our observations were made under drier and more continental weather in spring and summer.

We also pinpointed two other loci on chromosomes 1 and 5 using ‘PLov2-2n’ associated with SE that colocalized with QTLs identified by Crump *et al*. [14] using the same reference genome: chromosome 1, 13.5–21.0 Mbp (18.1 Mbp in our study); and chromosome 5, 12.9–15.5 Mbp (14.7 Mbp in our study). Interestingly, for PI we also found an SNP on chromosome 1 using ‘PLov2-2n’ (13.7 Mbp) colocalizing with the aforementioned QTL identified by Crump *et al*. [14]. Even if Crump *et al*. [14] determined the incidence of cracking without specifying the location of cracking, it could be hypothesized that Crump *et al*. [14] observed a sufficiently high amount of cracked fruit at different positions, and in particular at PI or SE, so that several of their significant associations for ‘general’ cracking tolerance correspond in fact to specific loci related to either PI or SE, as highlighted in our study and in Quero-García *et al*. [18]. Moreover, the search for colocalizations from the literature permitted us to identify additional ones, confirming the reliability of several marker–trait associations in this study concerning cracking-related traits.

Finally, the comparison of potentially colocalizing loci from the literature showed that, in most cases, the surrounding genes have the same annotation, even if they are physically distant. This would mean that searching for colocalization using physical positions may be meaningless if different genomes have been used to conduct the analyses. Even if a high level of synteny is expected between two genomes of the same species, the structural variations may explain such differences in physical positions.

### The molecular processes involved in sweet cherry fruit quality may be conserved in fruit species

A large proportion of the candidate genes identified are expected to be involved in fruit quality. We identified two genes encoding 2-oxoglutarate and Fe(II)-dependent oxygenase proteins at two different loci potentially involved in FW. In tomato, these proteins play crucial roles in the biosynthesis of steroidal glycoalkaloids, of polyphenol class of flavonoids, and of hormones such as gibberellins and ethylene during tomato growth and ripening [61]. This may explain why we also found ethylene metabolism-related genes linked with fruit size, such as a putative ethylene-responsive binding factor-associated repression ninja family and an ethylene-responsive transcription factor. Moreover, abscisic acid is another hormone known to be involved in fruit ripening [62, 63] and we identified a gene encoding an ABI 5 binding protein known to be involved in strawberry development and ripening [64].

For FW, we also identified a gene encoding a glucan endo-1,3-β-glucosidase, and such glycosyl hydrolases are enzymes linked to cell wall polysaccharide regulation [65] and cell wall remodeling [66, 67] during fruit ripening. Holušová *et al*. [26] also identified a gene encoding a β-glucosidase as a candidate gene for fruit size, making it a strong candidate gene. The fasciclin-like arabinogalactan proteins are also involved in cell wall biosynthesis in hemp and cotton [68, 69], and we identified a gene encoding its precursor associated with FWi and FT. Finally, we highlighted various transcription factors, such as MADS-box, MYB, and Hap3/NF-YB, that are known to be involved in fruit ripening, as in strawberry [70], banana [71], papaya [72], apricot [73], and tomato [74]. Similarly, Holušová *et al*. [26] also pinpointed genes encoding a cell-wall-associated receptor kinase and an endoglucanase, showing that the cell wall is undoubtedly directly involved in fruit size. More surprisingly, similarly to Holušová *et al*. [26], we did not identify any gene involved in cell number regulation associated with global fruit size, although it was identified by De Franceschi *et al*. [15].

Concerning fruit cracking, we identified a gene encoding a cyclin-dependent kinase inhibitor protein, which is usually considered an important component of cell cycle arrest in senescent and quiescent cells [75]. We also identified a programmed cell death regulatory protein that was shown to be involved in pear russet fruit skin [76]. In various fruits, including litchi, pomegranate and tomato, transcriptomics studies highlighted that the decomposition of pectin and the subsequent cell wall disassembly are crucial mechanisms leading to fruit cracking [77–79]. We found candidate genes encoding a putative xylogalacturonan β-1,3-xylosyltransferase, a probable galacturonosyltransferase-like, and a pectin methylesterase that are all linked to pectin metabolism and may play a role in the integrity of the sweet cherry cell wall [80]. Calcium is also an important nutrient in plants and has a role in cell wall integrity in case of disturbed metabolism [81, 82]. We found several candidate genes involved in calcium metabolism that may explain differences in susceptibility in SE, such as genes encoding a calcium-dependent protein kinase, a putative calcium-transporting ATPase, and probable calcium-binding protein. Our results are in line with the work of Wang *et al*. [78] in litchi, where calcium-related genes were downregulated in a cracking-susceptible cultivar compared with a resistant one. The same authors also found downregulation of MYB and AP transcription factors in the susceptible cultivar, and we identified both candidate genes. Moreover, the identification of a gene encoding a SAUR-like auxin-responsive protein tends to show that auxin could be involved in fruit cracking, maybe through cell wall acidification activity, as reported in Stortenbeker and Bemer [83]. Finally, we identified a gene encoding an asparagine synthetase and such an enzyme is expected to be involved in sweet cherry cracking [84]. To go further concerning fruit cracking, we looked for SNPs within coding sequences of genes that have been demonstrated to be involved in cell wall and cuticle formation and possibly with cracking, such as lipid transfer protein (*PaLTPG1*) and 3-ketoacyl-CoA synthase (*PaKCS6*) genes [85, 86]. We did identify variations in these genes in our population; however, these SNPs were not linked significantly to the variation in cracking-related traits.

If abscisic acid has a preponderant role in the development of non-climacteric fruits such as sweet cherry [63], our study tends to show that other hormones are probably also crucial for this physiological process, such as auxins [87], gibberellins, and, more surprisingly, ethylene. Together with hormones and primary metabolisms, fruit quality may be dependent on cell wall integrity, calcium response, and transcription factors. At the crossroads of these metabolisms, MATE transporters have an essential role, e.g. in flavonoid and phytohormone transport [88], and we found one MATE-encoding gene, together with other sugar and solute transporters, as global fruit size candidate genes. Finally, we identified several candidate genes, such as genes encoding a β-galactosidase, another cell wall hydrolytic enzyme, for an MYB, for a calcium-binding protein, and for a RLK/Pelle family of kinases, similar to genes potentially involved in global nut-size-related traits in walnut [47]. In summary, global fruit quality in sweet cherry could be partly the result of fine tuning of hormones, calcium, and cell wall metabolisms, and metabolite transporters, as already observed in other fruits.

### Conclusions

Although much is already known about sweet cherry fruit quality, there is still need for better understanding of the genetic control of such traits, in the context of breeding strategies for producer and consumer demands. This study provides the first attempt to evaluate genome-wide SNP–trait associations in sweet cherry for fruit quality traits, including fruit size, fruit firmness, and fruit cracking, using multiple reference genomes and multiple years of phenotyping. We successfully applied the GBS approach to generate a powerful set of SNPs that covered the entire cherry genome, and several SNP–trait associations related to different agronomically relevant traits were detected. We confirmed previously identified major loci involved in global fruit size and fruit cracking, together with numerous new loci, which constitutes the first step in the development of marker-assisted selection, in order to facilitate sweet cherry breeding. Finally, we pinpointed potential candidate genes mainly involved in hormone and cell wall metabolisms. Interestingly, our study showed the importance of the choice of the reference genome used to conduct a GWAS, together with consideration or not of MAF filtering.

## Materials and methods

### Plant material

Plant material consisted of a panel of 116 accessions belonging to the sweet cherry collection maintained by the INRAE *Prunus* Genetic Resources Center at Bourran (Lot and Garonne, France). The panel was chosen to (i) include the maximum genetic diversity from a collection of 210 accessions based on a previous study [45], and (ii) cover a wide range of phenotypic variability in fruit quality traits. The panel gathered accessions from France and other 15 countries in America, Asia, and Europe. It comprised old landraces and cultivars released by breeding programs more or less recently. The French accessions represented about half (48%) of the total panel, 78% of them being old landraces from different regions of the country (Supplementary Data Table S6).

### Phenotyping and phenotype modeling

Fruits were collected at maturity, randomly from one tree per accession. Assessment of ripening was subjective and involved mainly skin color, texture, and taste. A summary of the 23 traits evaluated is provided in Supplementary Data Table S7. All fruit traits were evaluated during the harvest day for each accession, on a homogenous sample of 10 fruits except for fruit-cracking-related traits, which were evaluated on 50 fruits. Each fruit in each sample was identified with a code number in order to score individually the data for each fruit and its corresponding stem and stone. Traits were measured with digital tools [balance for fruit weight (FW) and caliper for fruit height (FH), width (FWi), and thickness (FT)] and then averaged. Fruit firmness (FF) was measured using a Durofel^®^ (Setop Giraud Technologie, Cavaillon, France) texture analyzer as described by Campoy *et al*. [11]. After removing the stone from the fruits of each accession, fruits were pooled and the fruit juice was extracted with a hand press. Soluble sugar content (SSC) of the juice was measured with a digital refractometer (Atago Co., Ltd, Tokyo, Japan). The pH and titratable acidity (TA) were evaluated by using an equal volume of juice from 10 fruits as described by Dirlewanger *et al*. [89].

Fruit pistillar end cracking (PI), fruit stem end cracking (SE), and fruit side cracking (FS) were evaluated from a homogenous batch of 50 fruits. Each fruit was visually inspected and any observable crack was recorded by differentiating between three distinct fruit regions [18]. As proposed by these last authors, cracking tolerance was computed with percentages of count data, *a priori* following a multinomial distribution. Hence, an arcsine (square root) transformation was applied in order to stabilize the variance and to estimate genetic variances and heritabilities.

Fruit cracking susceptibility (FCS), fruit skin color (FC), and fruit juice color (FJC) were evaluated visually, according to the recommendations of the European Cooperative Programme for Plant Genetic Resources (ECPGR) *Prunus* working group (European *Prunus* Data Base: http://www.bordeaux.inra.fr/eucherrydb/), following an ordinal scale from 1 to 9. The Guidelines for the Conduct of Tests for Distinctness, Uniformity and Stability for sweet cherry recommended by the International Union for the Protection of New Varieties of Plants (UPOV) were used to determine fruit pistil end (FPE), fruit suture (FSU), and stone shape in central view (SS), following an ordinal scale from 1 to 3. Finally, productivity (PROD) was estimated according to an ordinal scale from 0 to 9, following the recommendations of INRAE Plant Genetic Resources Information System (Siregal) for sweet cherry (https://urgi.versailles.inrae.fr/siregal/siregal/grc.do). Nine traits were evaluated during 6 years (2014–19) and the remaining traits during 2 years (2014–15). Each year of evaluation was performed by a unique pair of evaluators.

The phenotypic data were analyzed using the ‘lme4’ R package [90]. For each trait, the means of genotypic effects were obtained for each accession using BLUPs as mixed linear models and by adjusting for the random year effect (2 or 6 years depending on the trait), as described in Bernard *et al*. [47].

The variance components were then used to estimate broad-sense heritability (*H*^2^) for all traits and Pearson correlation coefficients of each pair of the analyzed traits were calculated using the BLUP values with the ‘corrplot’ R package.

### DNA isolation, genotyping-by-sequencing library construction, and sequencing

Genomic DNA was extracted from young leaf tissues using the DNeasy Plant Mini Kit (Qiagen, Hilden, Germany). The DNA quality and concentration were measured with a NanoDrop™ 2000 spectrophotometer (Thermo Fisher Scientific, Waltham, USA) and a Qubit^®^ 2.0 fluorometer (Thermo Fisher Scientific, Waltham, USA). GBS library construction for multiplexed individuals was done at the CIRAD genotyping platform, Montpellier, France (https://www.gptr-lr-genotypage.com), according to a protocol adapted from Elshire *et al*. [27] with the use of the ApeKI restriction enzyme. Subsequently, the final pooled library was transferred to the GeT-PlaGe core facility (GenoToul GIS, Toulouse, France, https://get.genotoul.fr/) for sequencing using the Illumina^®^ paired-end protocol on a single lane in a HiSeq3000 sequencer (2 × 150 cycles).

### Genotyping-by-sequencing data handling, processing, and SNP calling

Raw fastq reads of all accessions were split into separate fastq files, based on their barcodes, using Sabre software (https://github.com/najoshi/sabre). The read quality of each fastq was checked with FastQC v.0.11.2 [91] and visualized with MultiQC v.1.7 [92]. All unligated adapters and low-quality read sequences were trimmed with Trimmomatic version 0.33 [93] with the following options: ILLUMINACLIP 2:30:10, LEADING 3, TRAILING 3, SLIDINGWINDOW 4:15, MINLEN 36. The trimmed and cleaned fastq files sequences were aligned to two of the available sweet cherry genome sequences, *P. avium* var. ‘Regina’ [37] and *P. avium* var. ‘Satonishiki’ [38], and the peach genome sequence *P. persica* var. ‘PLov2-2n’ v2 [36], using the Burrows–Wheeler Aligner 0.7.5a-r405 [93] and SAMtools [95]. The peach genome was used because of the high quality of both structural and functional annotations and the high synteny existing among *Prunus* species.

SNPs were called using the HaplotypeCaller algorithm as recommended by the Genome Analysis Toolkit v.3.7–1 [96]. Several filters were applied to minimize the number of false-positive SNPs using VCFtools 0.1.14 [97]. Only SNPs with all the following characteristics were retained for analysis: (i) missing data <20%; (ii) only one alternative allele; (iii) minor allele frequency (MAF) not less than 5%; (iv) minimum read depth of 10; and (v) a Phred scale mapping quality >60 (QUAL value >60). For some traits, we conducted analyses including the SNPs with MAF <5%. These analyses concerned the traits for which we found significant associations in GWAS analyses with the MAF filter to compare the results, and the traits that were highly inheritable for which we did not find significant associations with the MAF filter but for which known genomic regions were linked with in other studies.

Finally, we imputed the remaining 20% of missing data based on a matrix factorization approach using the ‘impute’ function from the ‘LEA’ R package [98]. Imputation of missing data is strongly recommended in genetic association studies [99] as it limits the discovery of false-positives.

### Population structure analysis and linkage disequilibrium decay

PCA was conducted with the ‘snpgdsPCA’ function of the ‘SNPRelate’ R package [100]. We also performed genetic clustering (structure) analysis using the sparse non-negative matrix factorization (sNMF) software, implemented in the ‘LEA’ R package [98]. For the sNMF analysis, 15 potential numbers (1–15) of ancestral populations (*K*) were tested using a cross-validation procedure, and the one with the lowest cross-entropy criterion error was chosen as the best *K* value [101].

LD decay was calculated with PopLDdecay v3.27 (https://github.com/BGI-shenzhen/PopLDdecay, accessed 28 October 2019) with SNPs with a maximum distance of 300 kb. LD decay was plotted as pairwise LD versus pairwise distance between SNP using the ‘smooth.spline’ function of the ‘stats’ R package.

### Genome-wide association analyses

The filtered SNP datasets from the 116 individuals of the sweet cherry panel, obtained after alignment with the three genomes, were used to perform a GWAS for the 25 traits studied. The GWAS was carried out by applying two different multilocus and mixed linear models implemented in the ‘GAPIT v3.0’ R package [102]: the multilocus mixed model (MLMM) of Segura *et al*. [103] and the Fixed and random model Circulating Probability Unification (FarmCPU) of Liu *et al*. [104]. These two models are known to increase the power of detecting significant marker–trait associations while controlling false-positives due to confounding factors [55]. Familial relatedness was accounted for using the VanRaden method [105] and population structure was accounted for using the ‘model selection’ option, which defines the best number of PC to include in the model according to a Bayesian information criterion (BIC), both implemented in GAPIT. Significant marker–trait associations were adjusted by multiple test correction using Bonferroni correction with an α value of 1% and were then inspected in Manhattan plots. The cutoff value (green solid line in the Manhattan plots) was calculated as −log_10_ (*α*/*k*), where *α* is the alpha value (0.01) and *k* is the number of SNPs. A quantile-quantile (Q–Q) plot was used to verify if the model was correctly accounting for confounding variables. We also conducted analyses including the SNPs with an MAF <5% for fruit size-related traits, cracking-related traits, and fruit firmness, to observe whether such filtering has a strong influence.

### Search for colocalizations of SNP–trait associations between reference genomes

As we performed GWAS using multiple reference genomes, we searched for potential colocalizations of SNP–trait associations that are relatively close in our results between two genomes, or close to those included in the results of nine previously published articles regarding fruit quality traits in sweet cherry. Consequently, we took sequences of 100–150 bp around the SNP associated with one trait of the first genome and we searched for the position of this sequence on the second genome. We then determined whether this sequence colocalized with the SNP associated with the same trait on the second genome, using the Basic Local Alignment Search Tool (BLAST) from the National Center for Biotechnology Information (NCBI) database (https://www.ncbi.nlm.nih.gov/).

### Search of candidate genes within the linkage disequilibrium blocks of associated loci

LD block analysis for the chromosomes identified with the SNPs significantly associated with a trait was performed using Haploview 4.2 software [106]. We obtained our Haploview files using PLINK software and the command ‘—recodeHV’ [107]. An exploration around each physical position of these SNP–phenotype associations was conducted to identify the other SNPs that are in linkage disequilibrium and to determine the genomic regions to search for candidate genes. Haplotype blocks in the region were defined with the ‘solid spine of LD’ method. The identified LD blocks were then searched for candidate genes using the ‘GFF3’ annotations of the three genomes.

## Acknowledgements

We thank the *Prunus/Juglans* Biological Resources Center managed by the INRAE Fruit Tree Experimental Unit for performing part of the phenotyping and for maintenance of the collection. This work was supported by the Région Nouvelle-Aquitaine with the project CerGEn reference 2018-1R20203, which funded the postdoctoral fellowship for A.S.L.D.

## Author contributions

E.D., T.B. and J.Q.G. designed the research. E.D. coordinated the research. H.B. and T.B. managed and performed the phenotyping. M.F. prepared the samples for GBS. A.S.L.D. performed bioinformatics analyses for GBS data. A.S.L.D. and A.B. performed GWAS and genomic analyses with the help of L.L.D. and B.W. T.B., J.Q.G. and ED worked on the interpretation of results. A.S.L.D. and A.B. wrote the manuscript. All coauthors revised and validated the manuscript.

## Data availability

The complete raw dataset can be freely and openly accessed in the ‘Recherche Data Gouv’ repository, via the identifier ‘GWAS on fruit quality traits using INRAE sweet cherry (*P. avium*) germplasm collection’ and at https://doi.org/10.57745/WVMJOV. The dataset consists of the list of plant material, genotyping resources, and phenotyping dataset.

## Conflict of interest

The authors declare that the research was conducted in the absence of any commercial or financial relationships that could be construed as a potential conflict of interest.

## Supplementary data

Supplementary data is available at *Horticulture Research* online.

## Supplementary Material

Web_Material_uhad191

## References

[1] Bujdoso G , Hrotko K. Cherry production. In: Quero-García J, Iezzoni A, Puławska J, Lang G, eds. Cherries: Botany, Production and Uses. CABI: Wallingford, 2017, 1–13

[2] Hedrick UP . The history of cultivated cherries. In: Hedrick UP, Howe GH, Taylor OM, Tubergen CB, Wellington R, eds. The Cherries of New York. J. B. Lyon: Albany, 1915,39–64

[3] Lee JC , Bruck DJ, Dreves AJ et al. In focus: spotted wing drosophila, *Drosophila suzukii*, across perspectives. *Pest Manag Sci*. 2011;67:1349–51 21990168 10.1002/ps.2271

[4] Quero-García J , Schuster M, López-Ortega G et al. Sweet cherry varieties and improvement. In: Quero-García J, Iezzoni A, Puławska J, Lang G, eds. Cherries: Botany, Production and Uses. CABI: Wallingford, 2017, 60–94

[5] Christensen JV . Rain-induced cracking of sweet cherries: its causes and prevention. In: Webster AD, Looney NE, eds. Cherries: Crop Physiology, Production and Uses. CABI: Wallingford, 1996,297–330

[6] Correia S , Schouten R, Silva AP et al. Sweet cherry fruit cracking mechanisms and prevention strategies: a review. *Sci Hortic*. 2018;240:369–77

[7] Knoche M , Winkler A. Rain-induced cracking of sweet cherries. In: Quero-García J, Iezzoni A, Puławska J, Lang G, eds. Cherries: Botany, Production and Uses. CABI: Wallingford, 2017, 140–65

[8] Yue C , Gallardo RK, Luby JJ et al. An evaluation of U.S. tart and sweet cherry producers trait prioritization: evidence from audience surveys. HortScience. 2014;49:931–7

[9] Tao R , Iezzoni AF. The S-RNase-based gametophytic self-incompatibility system in *Prunus* exhibits distinct genetic and molecular features. *Sci Hortic*. 2010;124:423–33

[10] Cai L , Quero-García J, Barreneche T et al. A fruit firmness QTL identified on linkage group 4 in sweet cherry (*Prunus avium* L.) is associated with domesticated and bred germplasm. Sci Rep. 2019;9:5008 30899090 10.1038/s41598-019-41484-8 PMC6428808

[11] Campoy JA , Le Dantec L, Barreneche T et al. New insights into fruit firmness and weight control in sweet cherry. *Plant Mol Biol Report*. 2015;33:783–96

[12] Calle A , Balas F, Cai L et al. Fruit size and firmness QTL alleles of breeding interest identified in a sweet cherry ‘Ambrunés’ × ‘Sweetheart’ population. Mol Breed. 2020;40:86

[13] Calle A , Wunsch A. Multiple-population QTL mapping of maturity and fruit-quality traits reveals LG4 region as a breeding target in sweet cherry (*Prunus avium* L.). Hortic Res. 2020;7:127 32821410 10.1038/s41438-020-00349-2 PMC7395078

[14] Crump WW , Peace C, Zhang Z et al. Detection of breeding-relevant fruit cracking and fruit firmness quantitative trait loci in sweet cherry via pedigree-based and genome-wide association approaches. *Front Plant Sci*. 2022;13:823250 35310633 10.3389/fpls.2022.823250 PMC8924583

[15] De Franceschi P , Stegmeir T, Cabrera A et al. Cell number regulator genes in *Prunus* provide candidate genes for the control of fruit size in sweet and sour cherry. Mol Breed. 2013;32:311–26 23976873 10.1007/s11032-013-9872-6 PMC3748327

[16] Rosyara UR , Bink MCAM, van de Weg E et al. Fruit size QTL identification and the prediction of parental QTL genotypes and breeding values in multiple pedigreed populations of sweet cherry. *Mol Breed*. 2013;32:875–87

[17] Zhang GR , Sebolt AM, Sooriyapathirana SS et al. Fruit size QTL analysis of an F-1 population derived from a cross between a domesticated sweet cherry cultivar and a wild forest sweet cherry. *Tree Genet Genomes*. 2010;6:25–36

[18] Quero-García J , Letourmy P, Campoy JA et al. Multi-year analyses on three populations reveal the first stable QTLs for tolerance to rain-induced fruit cracking in sweet cherry (*Prunus avium* L.). Hortic Res. 2021;8:136 34059661 10.1038/s41438-021-00571-6 PMC8166915

[19] Castède S , Campoy JA, Quero-García J et al. Genetic determinism of phenological traits highly affected by climate change in *Prunus avium*: flowering date dissected into chilling and heat requirements. *New Phytol*. 2014;202:703–15 24417538 10.1111/nph.12658

[20] Dirlewanger E , Quero-García J, Le Dantec L et al. Comparison of the genetic determinism of two key phenological traits, flowering and maturity dates, in three *Prunus* species: peach, apricot and sweet cherry. *Heredity*. 2012;109:280–92 22828898 10.1038/hdy.2012.38 PMC3477885

[21] Isuzugawa K , Shirasawa K, Kurosaka S et al. QTL analysis and candidate gene mapping for harvest day in sweet cherry (*Prunus avium* L.). Acta Hortic. 2019;1235:33–40

[22] Korte A , Farlow A. The advantages and limitations of trait analysis with GWAS: a review. *Plant Methods*. 2013;9:29 23876160 10.1186/1746-4811-9-29 PMC3750305

[23] Brachi B , Morris GP, Borevitz JO. Genome-wide association studies in plants: the missing heritability is in the field. *Genome Biol*. 2011;12:232 22035733 10.1186/gb-2011-12-10-232 PMC3333769

[24] Cortes LT , Zhang Z, Yu J. Status and prospects of genome-wide association studies in plants. *Plant Genome*. 2021;14:e20077 33442955 10.1002/tpg2.20077 PMC12806871

[25] Zhu C , Gore M, Buckler ES et al. Status and prospects of association mapping in plants. Plant Genome. 2008;1:5–20

[26] Holušová K , Čmejlová J, Suran P et al. High-resolution genome-wide association study of a large Czech collection of sweet cherry (*Prunus avium* L.) on fruit maturity and quality traits. Hortic Res. 2023;10:uhac233 36643756 10.1093/hr/uhac233 PMC9832837

[27] Elshire RJ , Glaubitz JC, Sun Q et al. A robust, simple genotyping-by-sequencing (GBS) approach for high diversity species. *PLoS One*. 2011;6:e19379 21573248 10.1371/journal.pone.0019379 PMC3087801

[28] Badenes ML , Fernández Martí A, Ríos G et al. Application of genomic technologies to the breeding of trees. *Front Genet*. 2016;7:198 27895664 10.3389/fgene.2016.00198 PMC5109026

[29] Imai A , Nonaka K, Kuniga T et al. Genome-wide association mapping of fruit-quality traits using genotyping-by-sequencing approach in citrus landraces, modern cultivars, and breeding lines in Japan. *Tree Genet Genomes*. 2018;14:24

[30] Lee SJ , Ban SH, Kim GH et al. Identification of potential gene-associated major traits using GBS-GWAS for Korean apple germplasm collections. *Plant Breed*. 2017;136:977–86

[31] Siddique MI , Lee H-Y, Ro N-Y et al. Identifying candidate genes for *Phytophthora capsici* resistance in pepper (*Capsicum annuum*) via genotyping-by-sequencing-based QTL mapping and genome-wide association study. *Sci Rep*. 2019;9:9962 31292472 10.1038/s41598-019-46342-1 PMC6620314

[32] Guajardo V , Solís S, Sagredo B et al. Construction of high density sweet cherry (*Prunus avium* L.) linkage maps using microsatellite markers and SNPs detected by genotyping-by-sequencing (GBS). PLoS One. 2015;10:e0127750 26011256 10.1371/journal.pone.0127750 PMC4444190

[33] Salazar JA , Pacheco I, Shinya P et al. Genotyping by sequencing for SNP-based linkage analysis and identification of QTLs linked to fruit quality traits in Japanese plum (*Prunus salicina* Lindl.). Front Plant Sci. 2017;8:476 28443103 10.3389/fpls.2017.00476 PMC5386982

[34] Elsadr H , Sherif S, Banks T et al. Refining the genomic region containing a major locus controlling fruit maturity in peach. *Sci Rep*. 2019;9:7522 31101872 10.1038/s41598-019-44042-4 PMC6525192

[35] Thurow LB , Gasic K, Raseira MCB et al. Genome-wide SNP discovery through genotyping by sequencing, population structure, and linkage disequilibrium in Brazilian peach breeding germplasm. *Tree Genet Genomes*. 2020;16:10

[36] Verde I , Jenkins J, Dondini L et al. The peach v2.0 release: high-resolution linkage mapping and deep resequencing improve chromosome-scale assembly and contiguity. *BMC Genomics*. 2017;18:225 28284188 10.1186/s12864-017-3606-9 PMC5346207

[37] Le Dantec L , Girollet N, Gouzy J et al. Assembly and annotation of 'Regina' sweet cherry genome. *Recherche Data Gouv*. 2020;V1 (1 March 2023, date last accessed)

[38] Shirasawa K , Isuzugawa K, Ikenaga M et al. The genome sequence of sweet cherry (*Prunus avium*) for use in genomics-assisted breeding. *DNA Res*. 2017;24:499–508 28541388 10.1093/dnares/dsx020 PMC5737369

[39] Muranty H , Troggio M, Sadok I et al. Accuracy and responses of genomic selection on key traits in apple breeding. Hortic Res. 2015;2:15060 26744627 10.1038/hortres.2015.60 PMC4688998

[40] Piaskowski J , Hardner C, Cai L et al. Genomic heritability estimates in sweet cherry reveal non-additive genetic variance is relevant for industry-prioritized traits. *BMC Genet*. 2018;19:23 29636022 10.1186/s12863-018-0609-8 PMC5894190

[41] Johnson DS . Influence of time of flower and fruit thinning on the firmness of ‘Cox’s Orange Pippin’ apples at harvest and after storage. *J Hortic Sci*. 1994;69:197–203

[42] Lerceteau-Kohler E , Moing A, Guerin G et al. Genetic dissection of fruit quality traits in the octoploid cultivated strawberry highlights the role of homoeo-QTL in their control. *Theor Appl Genet*. 2012;124:1059–77 22215248 10.1007/s00122-011-1769-3 PMC3304055

[43] Cantin CM , Gogorcena Y, Moreno AM. Phenotypic diversity and relationships of fruit quality traits in peach and nectarine *Prunus persica* (L.) Batsch breeding progenies. *Euphytica*. 2010;171:211–26

[44] Durel CE , Laurens F, Fouillet A et al. Utilization of pedigree information to estimate genetic parameters from large unbalanced data sets in apple. *Theor Appl Genet*. 1998;96:1077–85

[45] Campoy JA , Lerigoleur-Balsemin E, Christmann H et al. Genetic diversity, linkage disequilibrium, population structure and construction of a core collection of *Prunus avium* L. landraces and bred cultivars. BMC Plant Biol. 2016;16:49 26912051 10.1186/s12870-016-0712-9 PMC4765145

[46] Barreneche T , de la Cárcamo, Concepción M, Blouin-Delmas M et al. SSR-based analysis of genetic diversity and structure of sweet cherry (*Prunus avium* L.) from 19 countries in Europe. Plants (Basel). 2021;10:1983 34685793 10.3390/plants10101983 PMC8540667

[47] Bernard A , Crabier J, Donkpegan ASL et al. Genome-wide association study reveals candidate genes involved in fruit trait variation in Persian walnut (*Juglans regia* L.). Front Plant Sci. 2021;11:607213 33584750 10.3389/fpls.2020.607213 PMC7873874

[48] Arunyawat U , Capdeville G, Decroocq V et al. Linkage disequilibrium in French wild cherry germplasm and worldwide sweet cherry germplasm. *Tree Genet Genomes*. 2012;8:737–55

[49] Xanthopoulou A , Manioudaki M, Bazakos C et al. Whole genome re-sequencing of sweet cherry (*Prunus avium* L.) yields insights into genomic diversity of a fruit species. Hortic Res. 2020;7:60 32377351 10.1038/s41438-020-0281-9 PMC7193578

[50] Khatkar MS , Nicholas FW, Collins AR et al. Extent of genome-wide linkage disequilibrium in Australian Holstein-Friesian cattle based on a high-density SNP panel. *BMC Genomics*. 2008;9:187 18435834 10.1186/1471-2164-9-187 PMC2386485

[51] O’Brien AMP , Mészáros G, Utsunomiya YT et al. Linkage disequilibrium levels in *Bos indicus* and *Bos taurus* cattle using medium and high-density SNP chip data and different minor allele frequency distributions. *Livest Sci*. 2014;166:121–32

[52] Pightling AW , Petronella N, Pagotto F. Choice of reference sequence and assembler for alignment of *Listeria monocytogenes* short-read sequence data greatly influences rates of error in SNP analyses. *PLoS One*. 2014;9:e104579 25144537 10.1371/journal.pone.0104579 PMC4140716

[53] McCarthy M , Abecasis G, Cardon L et al. Genome-wide association studies for complex traits: consensus, uncertainty and challenges. *Nat Rev Genet*. 2008;9:356–69 18398418 10.1038/nrg2344

[54] McClure KA , Gardner KM, Douglas GM et al. A genome-wide association study of apple quality and scab resistance. Plant Genome. 2018;11:170075 10.3835/plantgenome2017.08.0075 PMC12962585 29505632

[55] Kaler A , Gillman J, Beissinger T et al. Comparing different statistical models and multiple testing corrections for association mapping in soybean and maize. *Front Plant Sci*. 2020;10:1794 32158452 10.3389/fpls.2019.01794 PMC7052329

[56] Valiente-Mullor C , Beamud B, Ansari I et al. One is not enough: on the effects of reference genome for the mapping and subsequent analyses of short-reads. *PLoS Comput Biol*. 2021;17:e1008678 33503026 10.1371/journal.pcbi.1008678 PMC7870062

[57] Gage JL , Vaillancourt B, Hamilton JP et al. Multiple maize reference genomes impact the identification of variants by genome-wide association study in a diverse inbred panel. *Plant Genome*. 2019;12:180069 10.3835/plantgenome2018.09.0069 PMC12810008 31290926

[58] Dirlewanger E , Graziano E, Joobeur T et al. Comparative mapping and marker-assisted selection in Rosaceae fruit crops. *Proc Natl Acad Sci USA*. 2004;101:9891–6 15159547 10.1073/pnas.0307937101 PMC470769

[59] Tabangin ME , Woo JG, Martin LJ. The effect of minor allele frequency on the likelihood of obtaining false positives. *BMC Proc*. 2009;3:S41 20018033 10.1186/1753-6561-3-S7-S41 PMC2795940

[60] Schumann C , Winkler A, Brüggenwirth M et al. Crack initiation and propagation in sweet cherry skin: a simple chain reaction causes the crack to 'run'. *PLoS One*. 2019;14:e0219794 31365556 10.1371/journal.pone.0219794 PMC6668808

[61] Wei S , Zhang W, Fu R et al. Genome-wide characterization of 2-oxoglutarate and Fe(II)-dependent dioxygenase family genes in tomato during growth cycle and their roles in metabolism. *BMC Genomics*. 2021;22:126 33602133 10.1186/s12864-021-07434-3 PMC7891033

[62] Gupta K , Wani SH, Razzaq A et al. Abscisic acid: role in fruit development and ripening. *Front Plant Sci*. 2022;13:817500 35620694 10.3389/fpls.2022.817500 PMC9127668

[63] Leng P , Yuan B, Guo YD. The role of abscisic acid in fruit ripening and responses to abiotic stress. *J Exp Bot*. 2014;65:4577–88 24821949 10.1093/jxb/eru204

[64] Li S , Li K, Ju Z et al. Genome-wide analysis of tomato NF-Y factors and their role in fruit ripening. *BMC Genomics*. 2016;17:36 26742635 10.1186/s12864-015-2334-2 PMC4705811

[65] Prasanna V , Prabha TN, Tharanathan RN. Fruit ripening phenomena – an overview. *Crit Rev Food Sci Nutr*. 2007;47:1–19 17364693 10.1080/10408390600976841

[66] Opassiri R , Pomthong B, Onkoksoong T et al. Analysis of rice glycosyl hydrolase family 1 and expression of Os4bglu12 β-glucosidase. *BMC Plant Biol*. 2006;6:33 17196101 10.1186/1471-2229-6-33 PMC1781453

[67] Schückel J , Kračun SK, Lausen TF et al. High-throughput analysis of endogenous fruit glycosyl hydrolases using a novel chromogenic hydrogel substrate assay. *Anal Methods*. 2017;9:1242–7

[68] Guerriero G , Mangeot-Peter L, Legay S et al. Identification of fasciclin-like arabinogalactan proteins in textile hemp (*Cannabis sativa* L.): in silico analyses and gene expression patterns in different tissues. *BMC Genomics*. 2017;18:741 28931375 10.1186/s12864-017-3970-5 PMC5606014

[69] Huang GQ , Gong SY, Xu WL et al. A fasciclin-like arabinogalactan protein, GhFLA1, is involved in fiber initiation and elongation of cotton. *Plant Physiol*. 2013;161:1278–90 23349362 10.1104/pp.112.203760 PMC3585596

[70] Pi M , Hu S, Cheng L et al. The MADS-box gene FveSEP3 plays essential roles in flower organogenesis and fruit development in woodland strawberry. *Hortic Res*. 2021;8:247 34848694 10.1038/s41438-021-00673-1 PMC8632884

[71] Choudhury SR , Roy S, Nag A et al. Characterization of an AGAMOUS-like MADS box protein, a probable constituent of flowering and fruit ripening regulatory system in banana. *PLoS One*. 2012;7:e44361 22984496 10.1371/journal.pone.0044361 PMC3439491

[72] Fu C , Chen H, Gao H et al. Two papaya MYB proteins function in fruit ripening by regulating some genes involved in cell-wall degradation and carotenoid biosynthesis. *J Sci Food Agric*. 2020;100:4442–8 32388883 10.1002/jsfa.10484

[73] Xi W , Feng J, Liu Y et al. The R2R3-MYB transcription factor PaMYB10 is involved in anthocyanin biosynthesis in apricots and determines red blushed skin. *BMC Plant Biol*. 2019;19:287 31262258 10.1186/s12870-019-1898-4 PMC6604168

[74] Li D , Mou W, Luo Z et al. Developmental and stress regulation on expression of a novel miRNA, Fan-miR73 and its target ABI5 in strawberry. *Sci Rep*. 2016;6:28385 27325048 10.1038/srep28385 PMC4914977

[75] Polymenis M , Kennedy BK. Unbalanced growth, senescence and aging. *Adv Exp Med Biol*. 2017;1002:189–208 28600787 10.1007/978-3-319-57127-0_8 PMC6345385

[76] Wang Y , Dai M, Wu X et al. An ARF1-binding factor triggering programmed cell death and periderm development in pear russet fruit skin. Hortic Res. 2022;9:uhab061 35043172 10.1093/hr/uhab061 PMC8947239

[77] Jiang F , Lopez A, Jeon S et al. Disassembly of the fruit cell wall by the ripening-associated polygalacturonase and expansin influences tomato cracking. Hortic Res. 2019;6:17 30729007 10.1038/s41438-018-0105-3 PMC6355925

[78] Wang J , Wu XF, Tang Y et al. RNA-Seq provides new insights into the molecular events involved in ‘ball-skin versus bladder effect’ on fruit cracking in litchi. *Int J Mol Sci*. 2021;22:454 33466443 10.3390/ijms22010454 PMC7796454

[79] Wang Y , Guo L, Zhao X et al. Advances in mechanisms and omics pertaining to fruit cracking in horticultural plants. *Agronomy*. 2021;11:1045

[80] Santos M , Egea-Cortines M, Gonçalves B et al. Molecular mechanisms involved in fruit cracking: a review. *Front Plant Sci*. 2023;14:1130857 36937999 10.3389/fpls.2023.1130857 PMC10016354

[81] Schumann C , Winkler A, Knoche M. Calcium decreases cell wall swelling in sweet cherry fruit. *Sci Rep*. 2022;12:16496 36192436 10.1038/s41598-022-20266-9 PMC9530156

[82] White PJ , Broadley MR. Calcium in plants. *Ann Bot*. 2003;92:487–511 12933363 10.1093/aob/mcg164 PMC4243668

[83] Stortenbeker N , Bemer M The SAUR gene family: the plant’s toolbox for adaptation of growth and development. J Exp Bot. 2019;70:17–27 30239806 10.1093/jxb/ery332

[84] Michailidis M , Karagiannis E, Bazakos C et al. Genotype- and tissue-specific metabolic networks and hub genes involved in water-induced distinct sweet cherry fruit cracking phenotypes. *Comput Struct Biotechnol J*. 2021;19:5406–20 34667535 10.1016/j.csbj.2021.09.030 PMC8501671

[85] Alkio M , Jonas U, Sprink T et al. Identification of putative candidate genes involved in cuticle formation in *Prunus avium* (sweet cherry) fruit. *Ann Bot*. 2012;110:101–12 22610921 10.1093/aob/mcs087 PMC3380588

[86] Balbontín C , Ayala H, Rubilar J et al. Transcriptional analysis of cell wall and cuticle related genes during fruit development of two sweet cherry cultivars with contrasting levels of cracking tolerance. *Chilean J Agric Res*. 2014;74:162–9

[87] Clayton-Cuch D , Yu L, Shirley N et al. Auxin treatment enhances anthocyanin production in the non-climacteric sweet cherry (*Prunus avium* L.). *Int J Mol Sci*. 2021;22:10760 34639100 10.3390/ijms221910760 PMC8509301

[88] Ku YS , Cheng SS, Cheung MY et al. The roles of multidrug and toxic compound extrusion (MATE) transporters in regulating agronomic traits. *Agronomy*. 2022;12:878

[89] Dirlewanger E , Moing A, Rothan C et al. Mapping QTLs controlling fruit quality in peach (*Prunus persica* (L.) Batsch). *Theor Appl Genet*. 1999;98:18–31

[90] Bates D , Maechler M, Bolker B et al. Fitting linear mixed-effects models using lme4. *J Stat Softw*. 2015;67:1–48

[91] Andrews S. FastQC: A Quality Control Tool for High Throughput Sequence Data. 2010. http://www.bioinformatics.babraham.ac.uk/projects/fastqc (1 August 2020, date last accessed)

[92] Ewels P , Magnusson M, Lundin S et al. MultiQC: summarize analysis results for multiple tools and samples in a single report. *Bioinformatics*. 2016;32:3047–8 27312411 10.1093/bioinformatics/btw354 PMC5039924

[93] Bolger AM , Lohse M, Usadel B. Trimmomatic: a flexible trimmer for Illumina sequence data. *Bioinformatics*. 2014;30:2114–20 24695404 10.1093/bioinformatics/btu170 PMC4103590

[94] Li H , Durbin R. Fast and accurate short read alignment with Burrows–Wheeler transform. *Bioinformatics*. 2009;25:1754–60 19451168 10.1093/bioinformatics/btp324 PMC2705234

[95] Li H , Handsaker B, Wysoker A et al. The sequence alignment/map format and SAMtools. *Bioinformatics*. 2009;25:2078–9 19505943 10.1093/bioinformatics/btp352 PMC2723002

[96] McKenna A , Hanna M, Banks E et al. The genome analysis toolkit: a MapReduce framework for analyzing next-generation DNA sequencing data. *Genome Res*. 2010;20:1297–303 20644199 10.1101/gr.107524.110 PMC2928508

[97] Danecek P , Auton A, Abecasis G et al. The variant call format and VCFtools. *Bioinformatics*. 2011;27:2156–8 21653522 10.1093/bioinformatics/btr330 PMC3137218

[98] Frichot E , François O. LEA: an R package for landscape and ecological association studies. *Methods Ecol Evol*. 2015;6:925–9

[99] Marchini J , Howie B. Genotype imputation for genome-wide association studies. Nat Rev Genet. 2010;11:499–511 20517342 10.1038/nrg2796

[100] Zheng X , Levine D, Shen J et al. A high-performance computing toolset for relatedness and principal component analysis of SNP data. *Bioinformatics*. 2012;28:3326–8 23060615 10.1093/bioinformatics/bts606 PMC3519454

[101] Frichot E , Mathieu F, Trouillon T et al. Fast and efficient estimation of individual ancestry coefficients. *Genetics*. 2014;196:973–83 24496008 10.1534/genetics.113.160572 PMC3982712

[102] Lipka AE , Tian F, Wang Q et al. GAPIT: genome association and prediction integrated tool. *Bioinformatics*. 2012;28:2397–9 22796960 10.1093/bioinformatics/bts444

[103] Segura V , Vilhjálmsson BJ, Platt A et al. An efficient multi-locus mixed-model approach for genome-wide association studies in structured populations. *Nat Genet*. 2012;44:825–30 22706313 10.1038/ng.2314 PMC3386481

[104] Liu X , Huang M, Fan B et al. Iterative usage of fixed and random effect models for powerful and efficient genome-wide association studies. *PLoS Genet*. 2016;12:e1005957 26828793 10.1371/journal.pgen.1005767 PMC4734661

[105] VanRaden PM . Genomic measures of relationship and inbreeding. *Interbull Bull*. 2007;37:33

[106] Barrett JC , Fry B, Maller J et al. Haploview: analysis and visualization of LD and haplotype maps. *Bioinformatics*. 2005;21:263–5 15297300 10.1093/bioinformatics/bth457

[107] Purcell S , Neale B, Todd-Brown K et al. PLINK: a tool set for whole-genome association and population-based linkage analyses. *Am J Hum Genet*. 2007;81:559–75 17701901 10.1086/519795 PMC1950838

