# Supplementary material for: Genome-wide association mapping in a sweet cherry germplasm collection (Prunus avium L.) reveals candidate genes for fruit quality traits
Source: Hortic Res. 2023 Sep 19;10(10):uhad191. doi: 10.1093/hr/uhad191 (PMC10794993; doi:10.1093/hr/uhad191)
Supplement: Web_Material_uhad191 [file web_material_uhad191.zip › Figure S3 - Population structure.pdf]

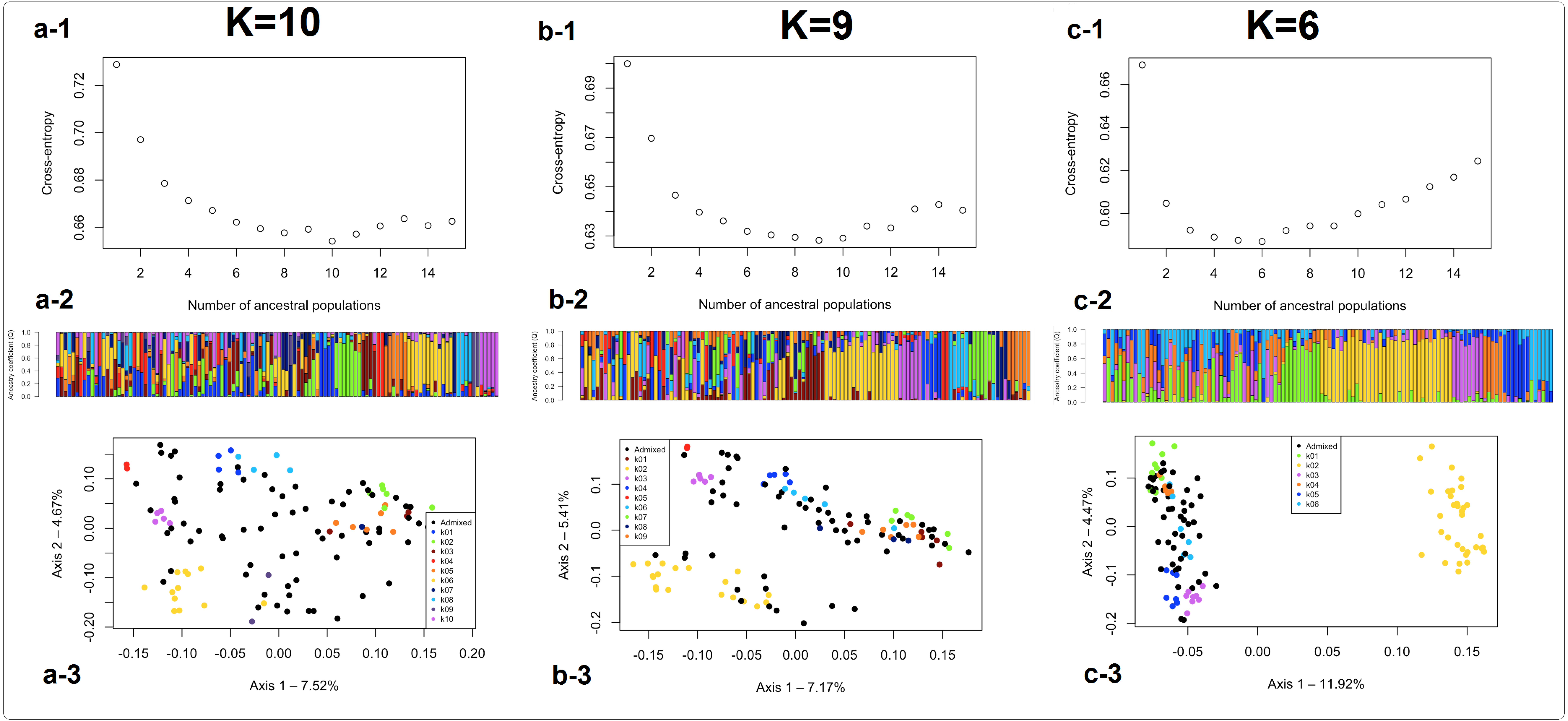

a-1
K=10
Cross-entropy
Number of ancestral populations
a-2
Ancestry coefficient (Q)
a-3
Axis 1 – 7.52%
Axis 2 – 4.67%
Admixed
k01
k02
k03
k04
k05
k06
k07
k08
k09
k10
b-1
K=9
Cross-entropy
Number of ancestral populations
b-2
Ancestry coefficient (Q)
b-3
Axis 1 – 7.17%
Axis 2 – 5.41%
Admixed
k01
k02
k03
k04
k05
k06
k07
k08
k09
c-1
K=6
Cross-entropy
Number of ancestral populations
c-2
Ancestry coefficient (Q)
c-3
Axis 1 – 11.92%
Axis 2 – 4.47%
Admixed
k01
k02
k03
k04
k05
k06
