# Supplementary material for: Genome-wide association mapping in a sweet cherry germplasm collection (Prunus avium L.) reveals candidate genes for fruit quality traits
Source: Hortic Res. 2023 Sep 19;10(10):uhad191. doi: 10.1093/hr/uhad191 (PMC10794993; doi:10.1093/hr/uhad191)
Supplement: Web_Material_uhad191 [file web_material_uhad191.zip › Figure S8 - Comparison MAF filtering for fruit weight.pdf]

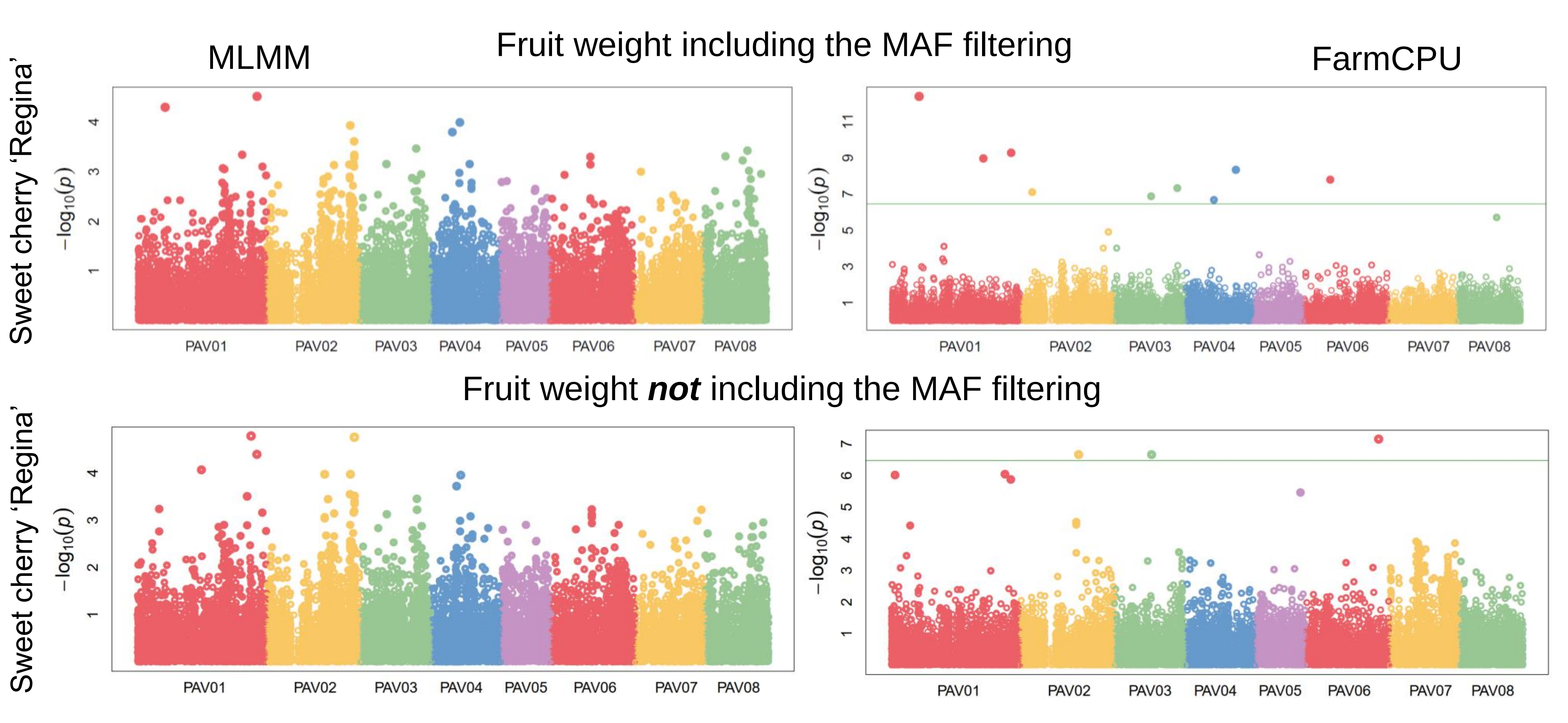

Fruit weight including the MAF filtering
MLMM
FarmCPU
Sweet cherry ‘Regina’
−log10(p)
PAV01
PAV02
PAV03
PAV04
PAV05
PAV06
PAV07
PAV08
Fruit weight not including the MAF filtering
Sweet cherry ‘Regina’
−log10(p)
