# Supplementary figures and images for: Genome-wide association mapping in a sweet cherry germplasm collection (Prunus avium L.) reveals candidate genes for fruit quality traits
Source: Hortic Res. 2023 Sep 19;10(10):uhad191. doi: 10.1093/hr/uhad191 (PMC10794993; doi:10.1093/hr/uhad191)

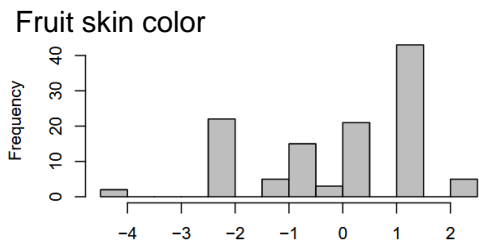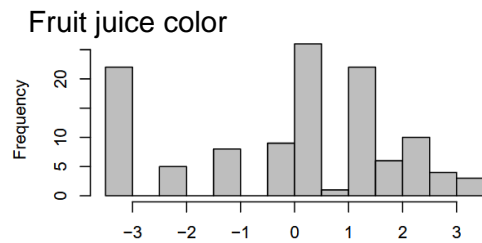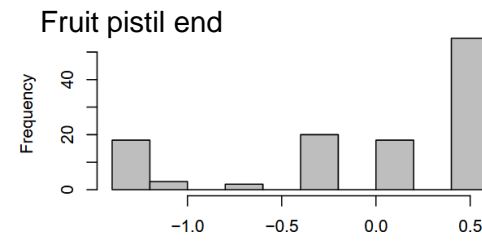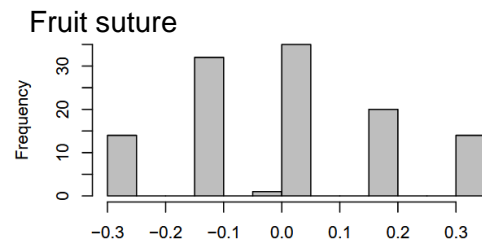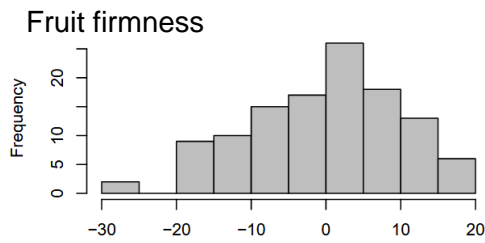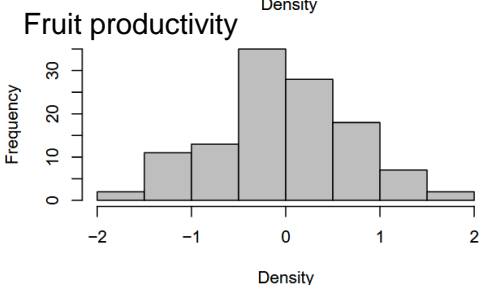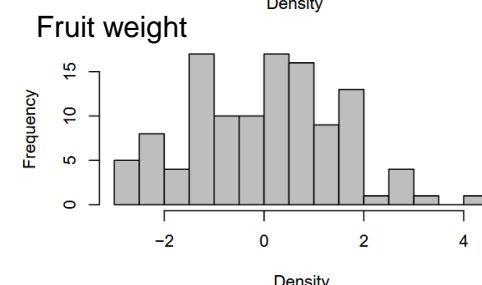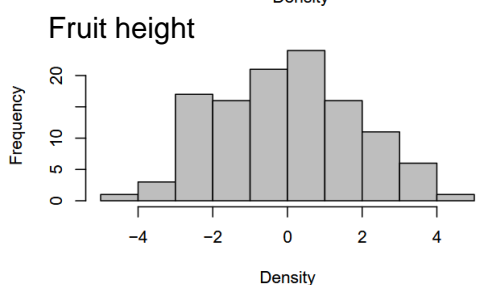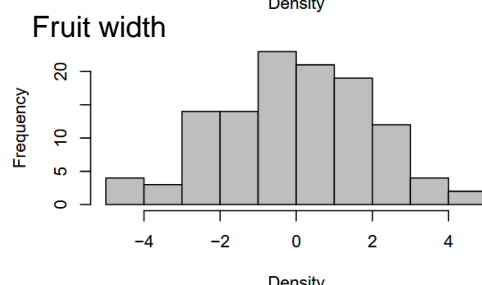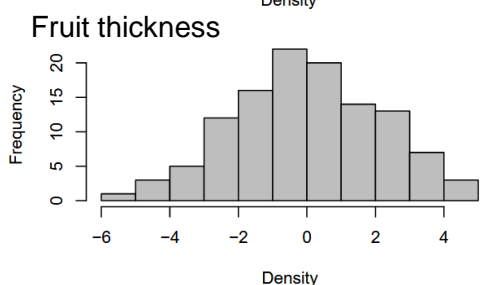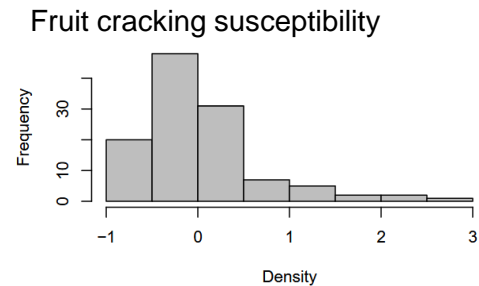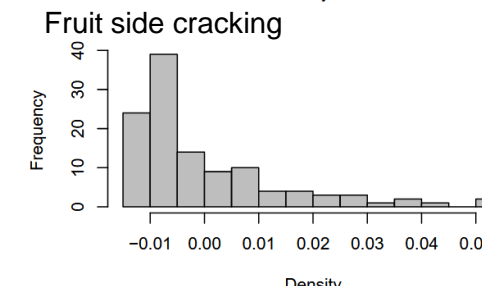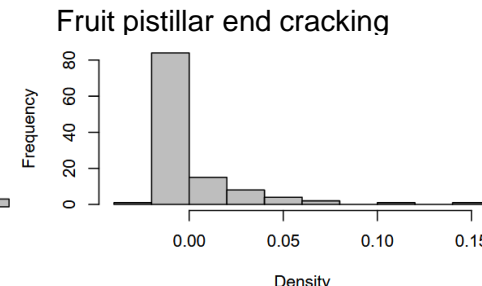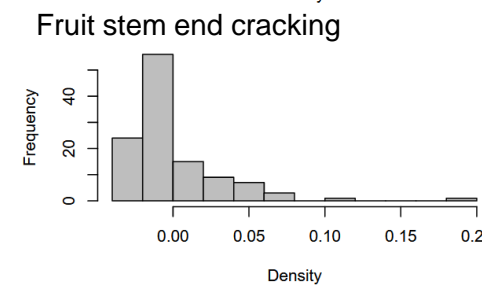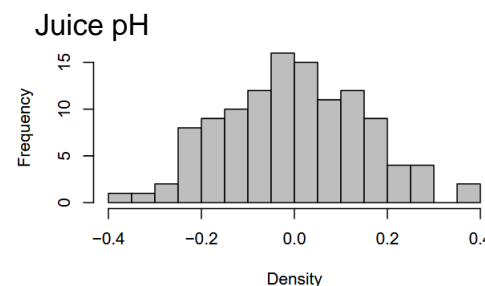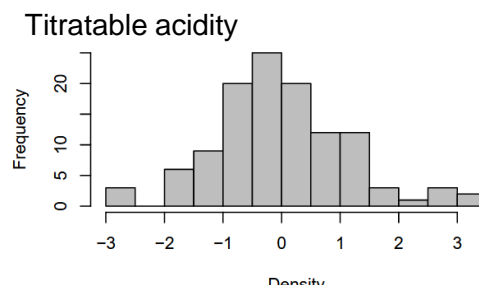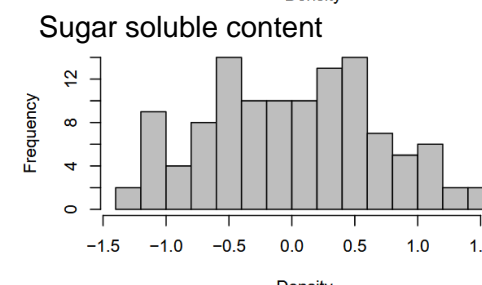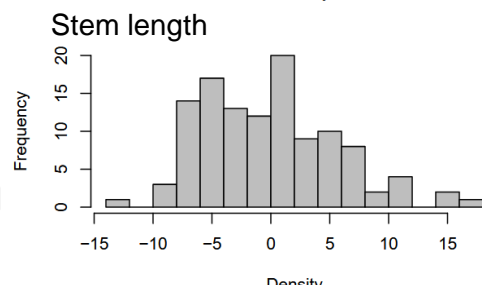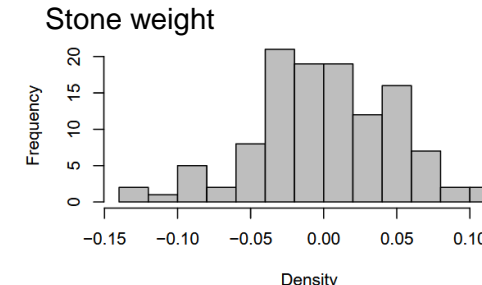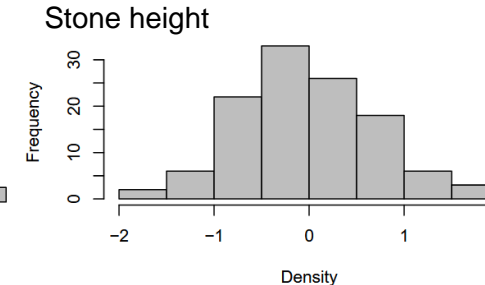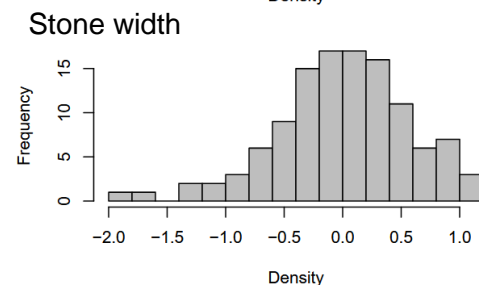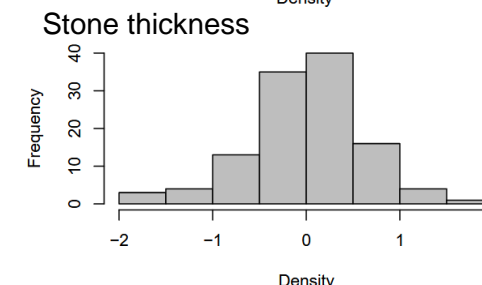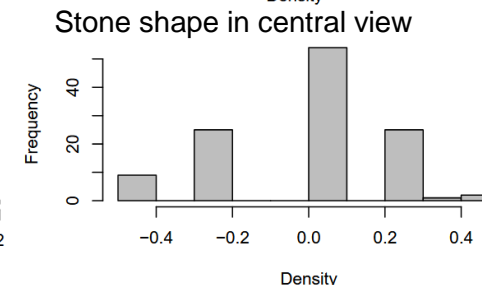

Supplement: Web_Material_uhad191 [file web_material_uhad191.zip › Figure S1 - Frequency distributions.pdf]

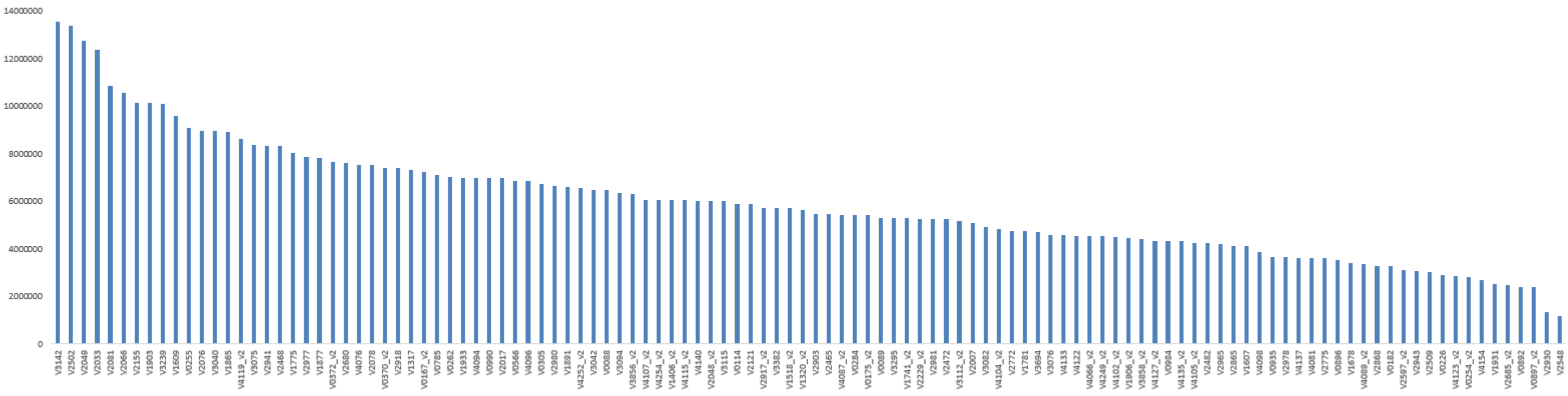

Supplement: Web_Material_uhad191 [file web_material_uhad191.zip › Figure S2 - Reads per sample.pdf]

## LD decay

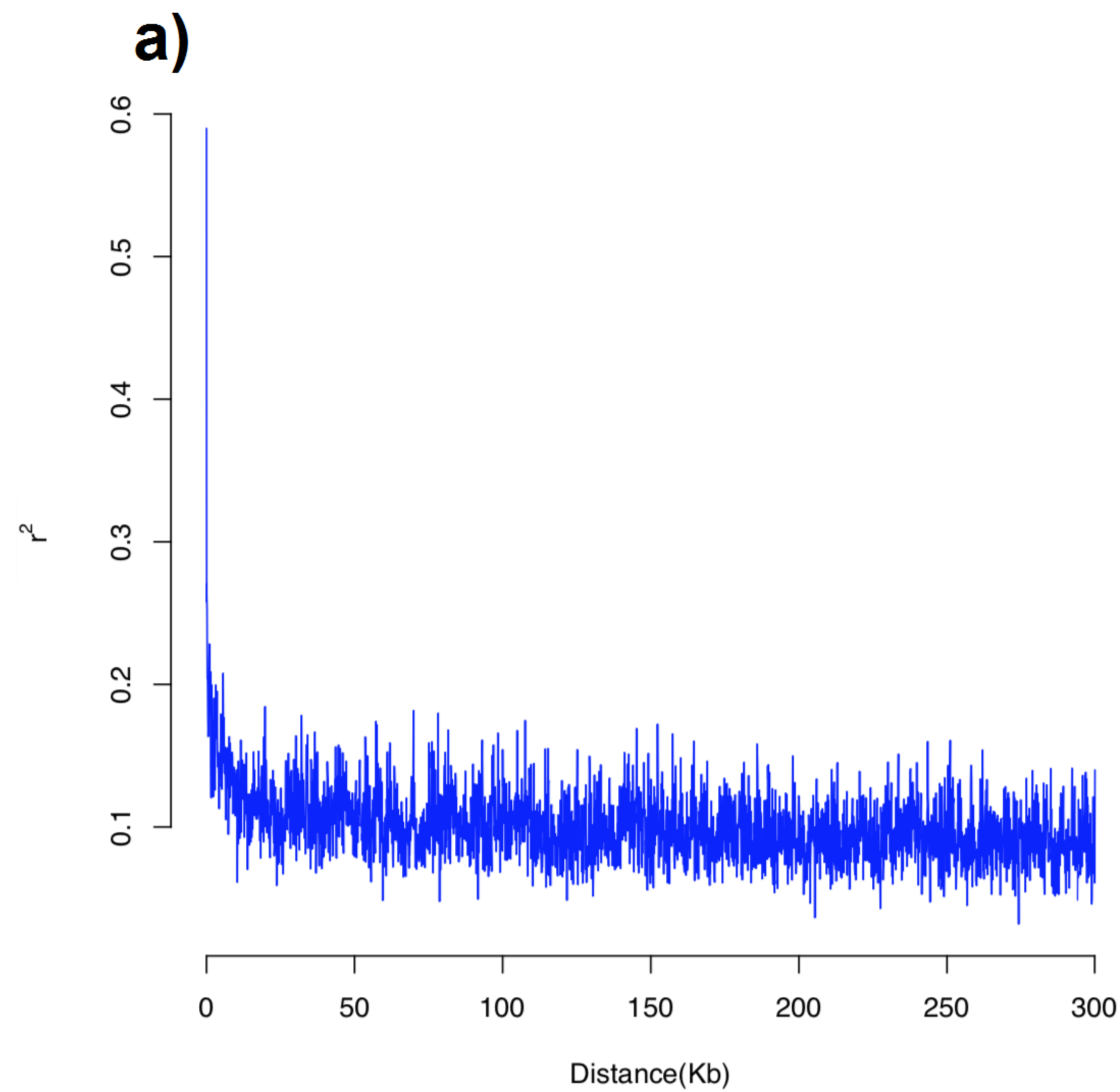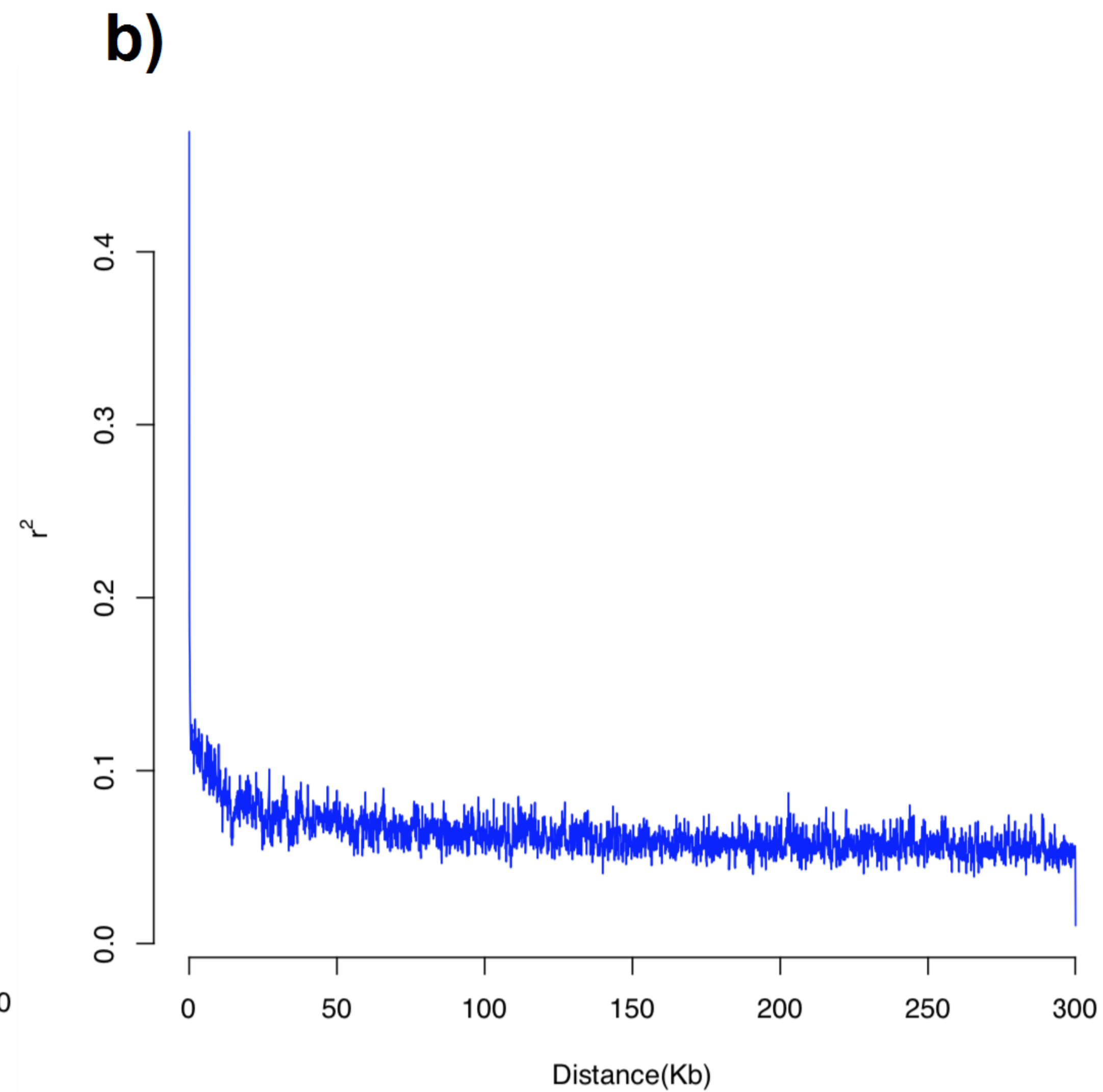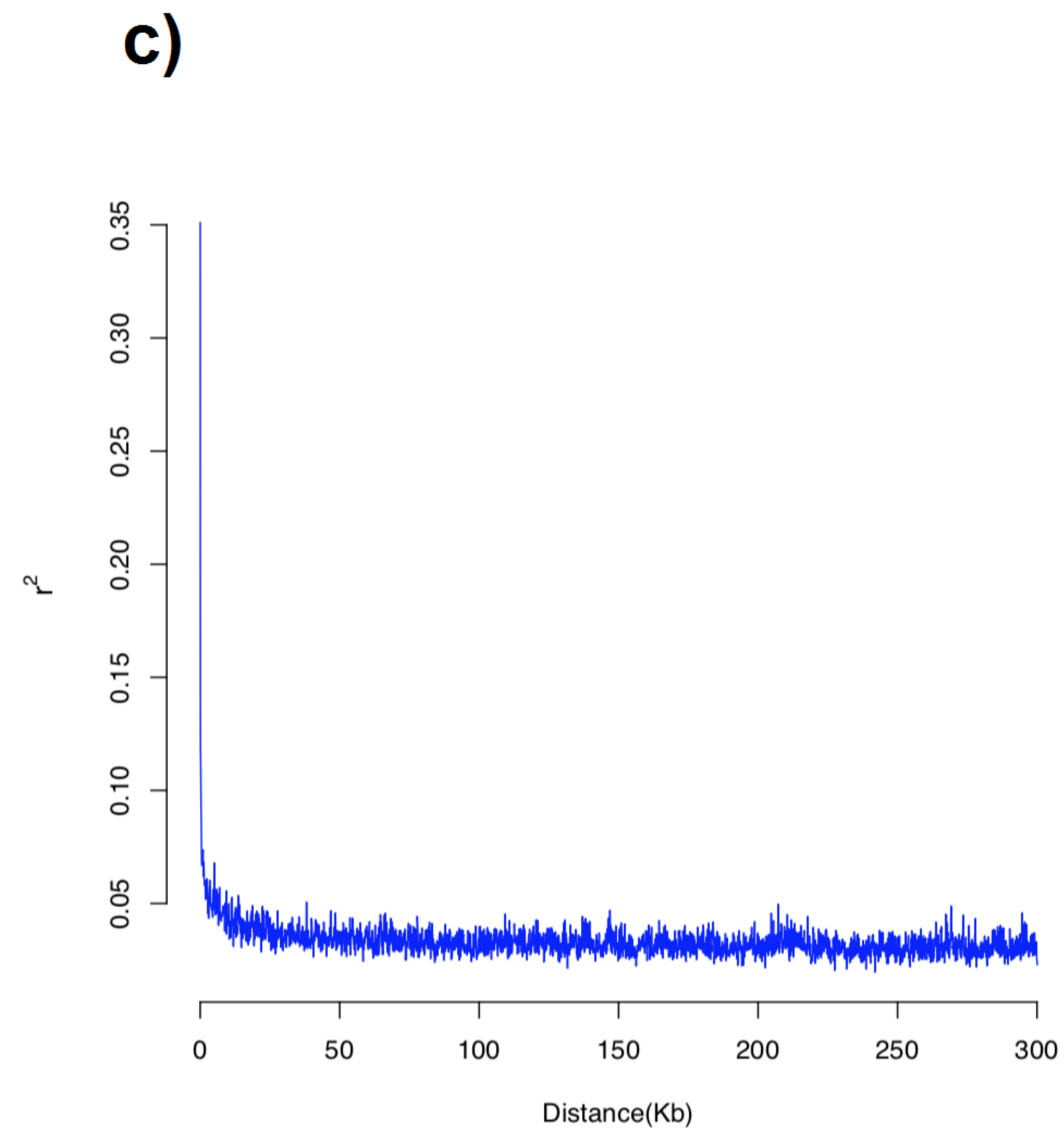

Supplement: Web_Material_uhad191 [file web_material_uhad191.zip › Figure S4 - LD decay.pdf]

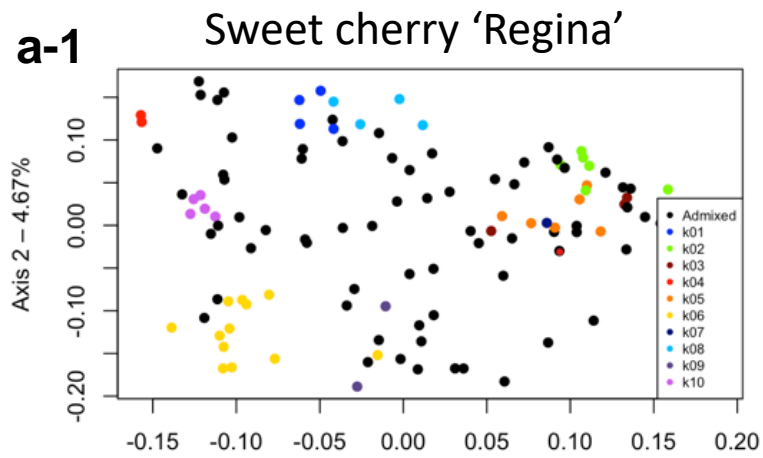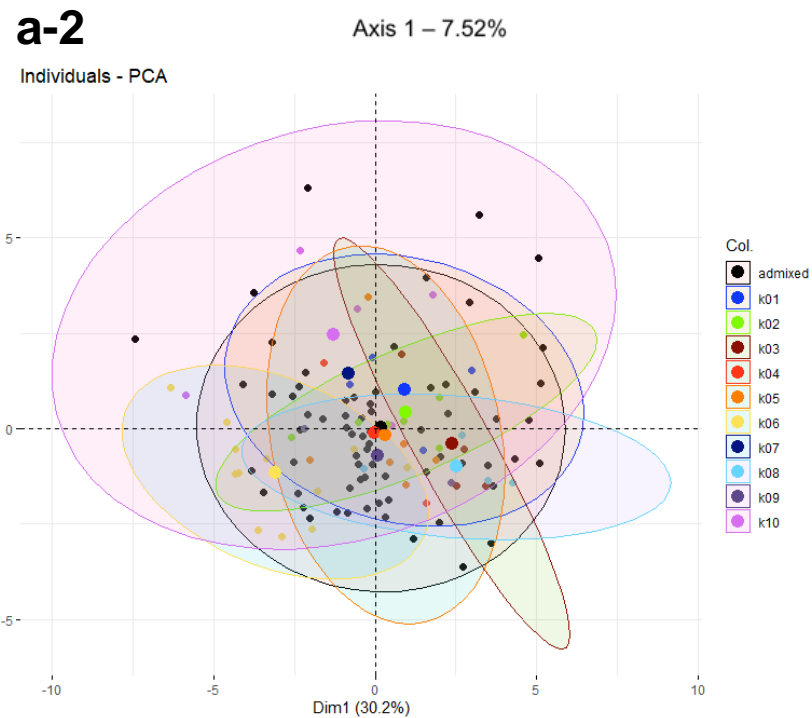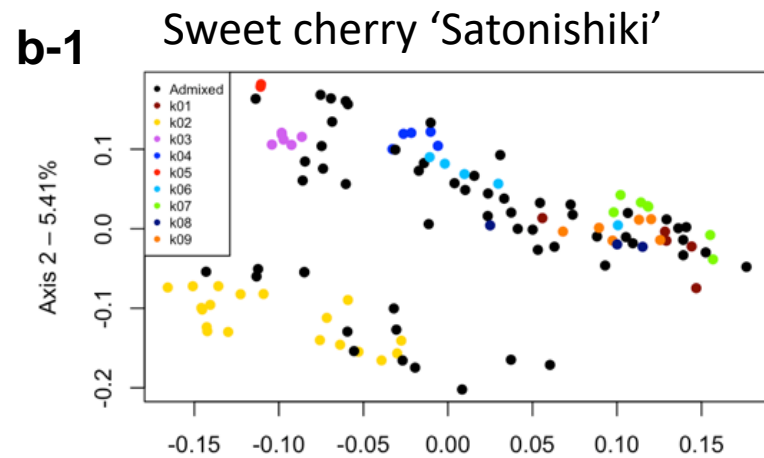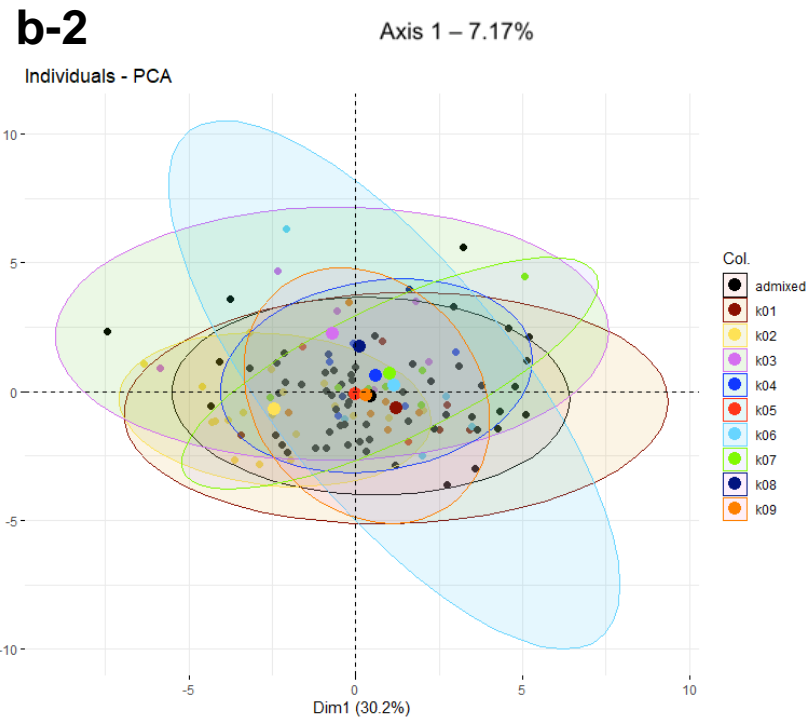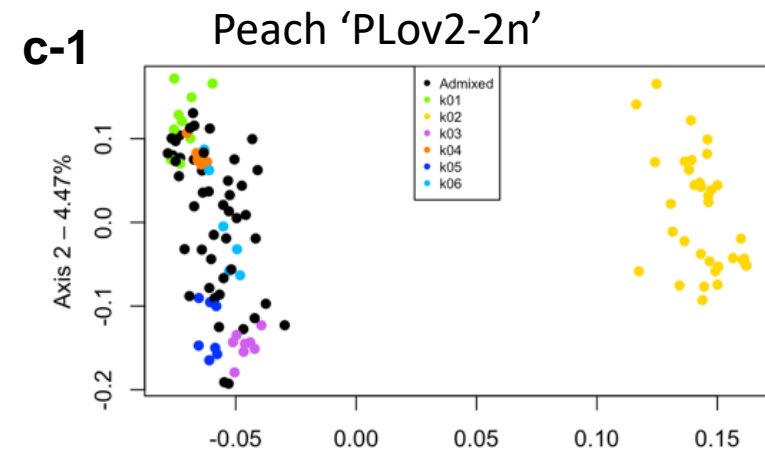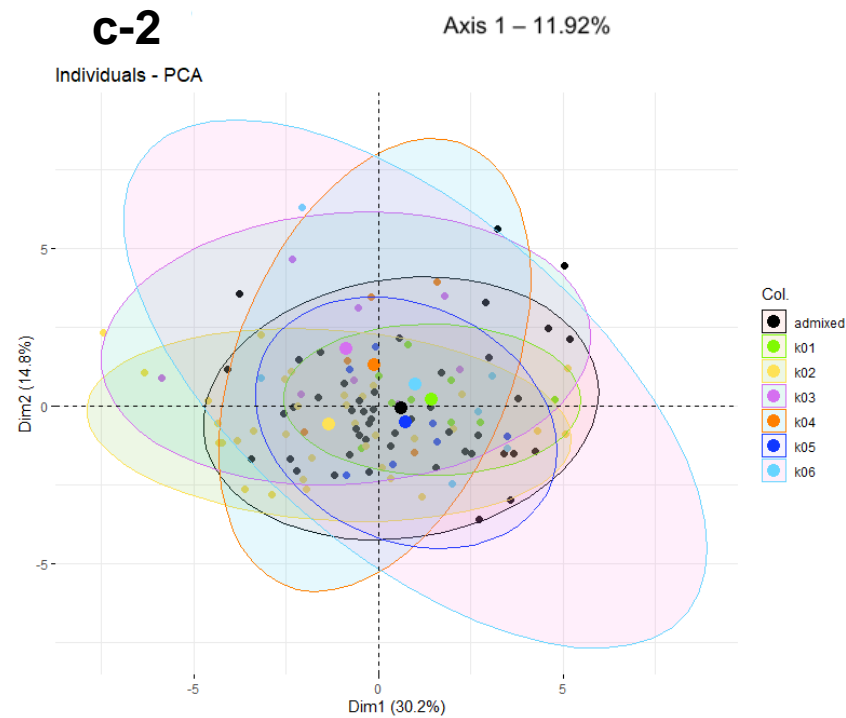

Supplement: Web_Material_uhad191 [file web_material_uhad191.zip › Figure S5 - PCA structure vs. BLUPs.pdf]

Fruit stem end cracking

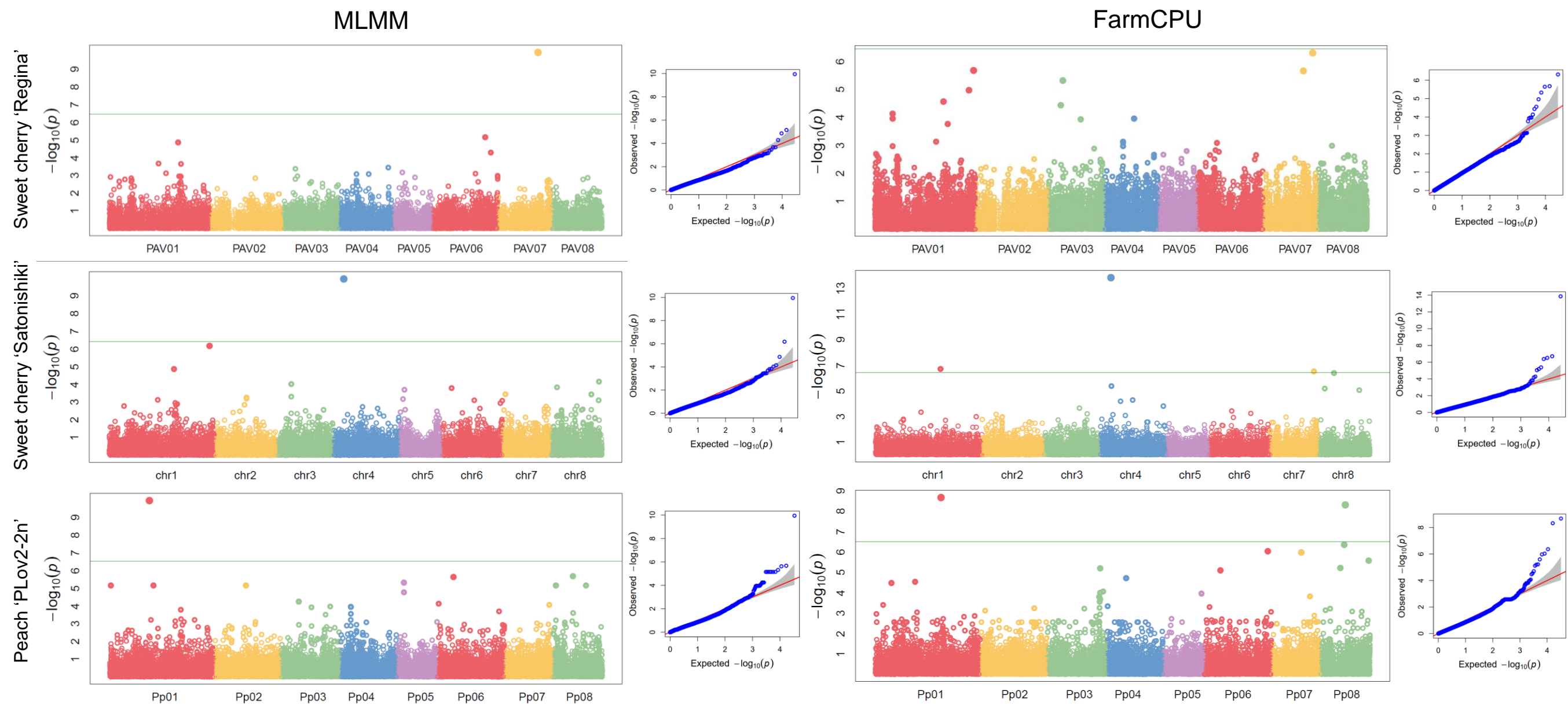

Fruit pistillar end cracking

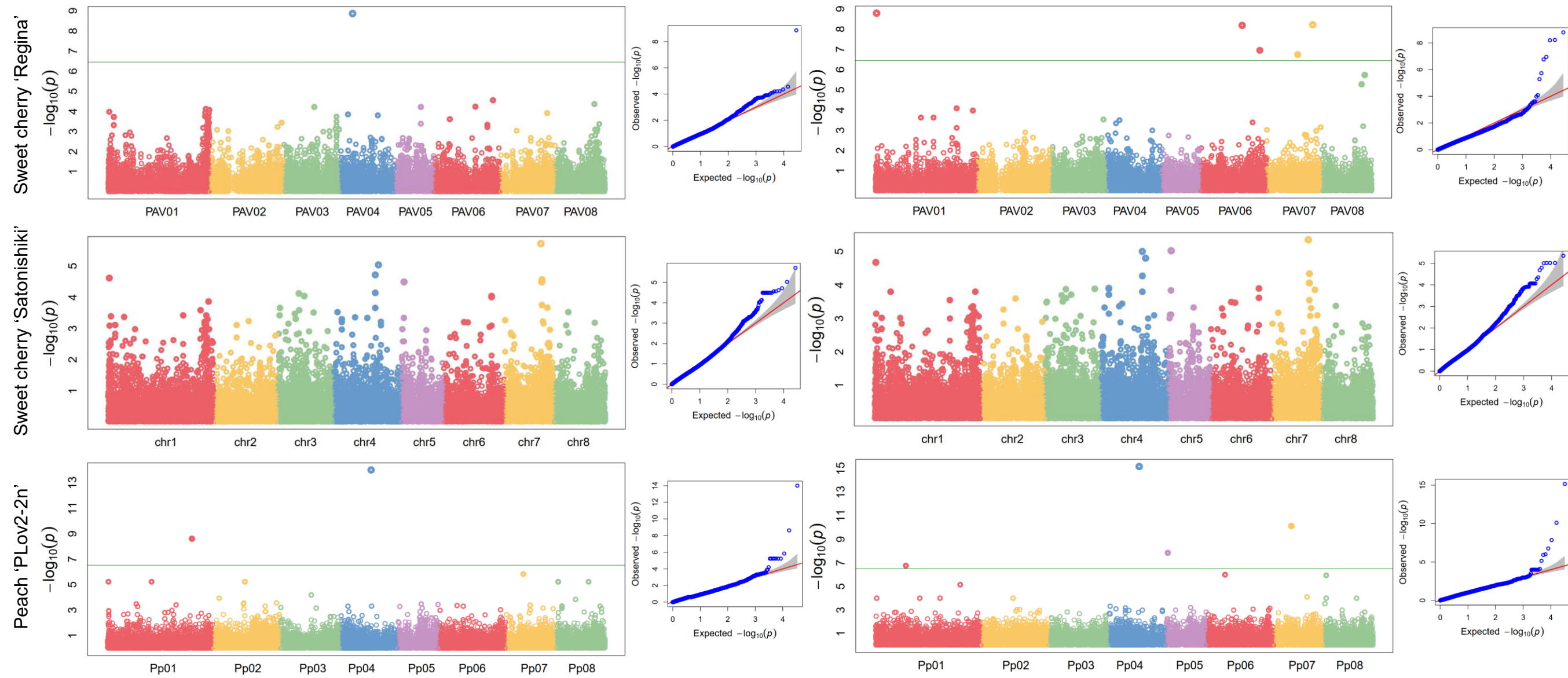

Supplement: Web_Material_uhad191 [file web_material_uhad191.zip › Figure S6 - GWAS for cracking-related traits.pdf]

MLMM

Stone width

FarmCPU

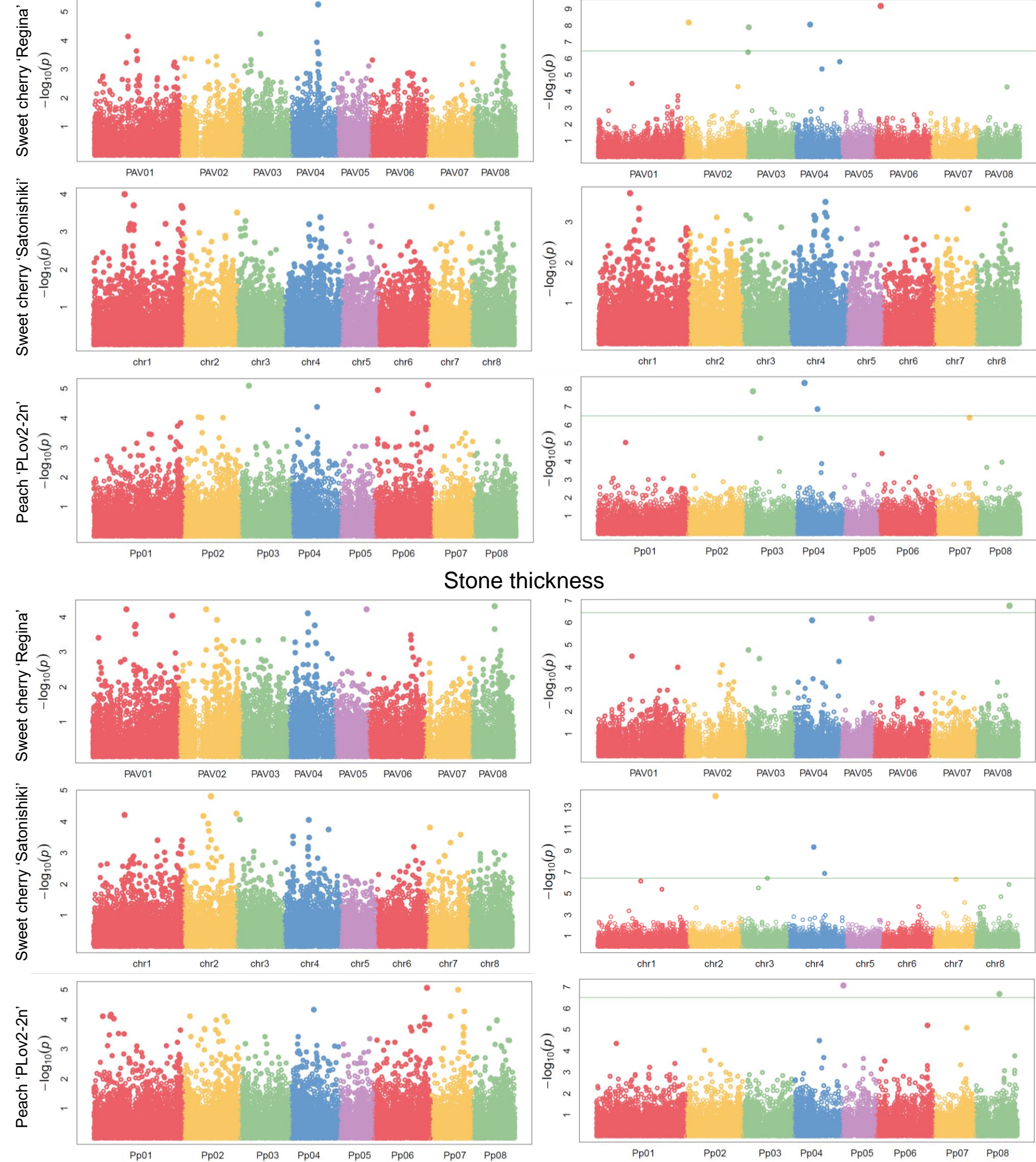

Supplement: Web_Material_uhad191 [file web_material_uhad191.zip › Figure S7 - GWAS for stone size.pdf]
